# Pocket Crafter: a 3D generative modeling based workflow for the rapid generation of hit molecules in drug discovery

**DOI:** 10.1186/s13321-024-00829-w

**Published:** 2024-03-21

**Authors:** Lingling Shen, Jian Fang, Lulu Liu, Fei Yang, Jeremy L. Jenkins, Peter S. Kutchukian, He Wang

**Affiliations:** Novartis Biomedical Research, Cambridge, MA 02139 USA

**Keywords:** Hit finding, Drug discovery, 3D generative chemistry, WDR5, Pocket Crafter

## Abstract

We present a user-friendly molecular generative pipeline called Pocket Crafter, specifically designed to facilitate hit finding activity in the drug discovery process. This workflow utilized a three-dimensional (3D) generative modeling method Pocket2Mol, for the de novo design of molecules in spatial perspective for the targeted protein structures, followed by filters for chemical-physical properties and drug-likeness, structure–activity relationship analysis, and clustering to generate top virtual hit scaffolds. In our WDR5 case study, we acquired a focused set of 2029 compounds after a targeted searching within Novartis archived library based on the virtual scaffolds. Subsequently, we experimentally profiled these compounds, resulting in a novel chemical scaffold series that demonstrated activity in biochemical and biophysical assays. Pocket Crafter successfully prototyped an effective end-to-end 3D generative chemistry-based workflow for the exploration of new chemical scaffolds, which represents a promising approach in early drug discovery for hit identification.

**Supplementary Information:**

The online version contains supplementary material available at 10.1186/s13321-024-00829-w.

## Scientific contribution

Hit identification is a time-consuming and costly step in drug discovery process. Here we developed a molecule generative pipeline called Pocket Crafter that can speed up this process greatly. This workflow utilized 3D generative modeling method Pocket2Mol for the de novo design of molecules in spatial perspective for the target and applies filters for chemical-physical properties and drug-likeness to generate top virtual hits with further structure–activity relationship analysis and clustering to output a focused set of hit compounds, which led to the success of hit finding as it showed in our demo case.

**Supplementary Information:**

The online version contains supplementary material available at 10.1186/s13321-024-00829-w.

## Introduction

Hit identification is an essential and challenging step in the drug discovery process due to limited understanding of disease biology or target complexity, as well as the constraints of screening assays [1]. High-throughput screening for hit compound identification, can be both costly and time-consuming [1], which limits the number of the potential targets that can be screened and the diversity of hit chemicals for each target. To address these challenges and expedite the drug development process, innovative computational tools are being extensively employed [2, 3]. While traditional structure-based virtual screening plays a vital role in identifying diverse hits through discriminative models, there is still considerable room for improvement in terms of hit rate and chemotype diversity in the early hit discovery stage [4].

Generative models offer a distinct approach by learning to represent and optimize molecules in a continuous latent space [5, 6]. They have proven to be highly effective in generating one-dimensional (1D) molecules with SMILES (Simplified molecular-input line-entry system) representations [7, 8] and two-dimensional (2D) molecules with molecular graphs representations [9]. This advancement holds the potential to accelerate the hit discovery process and minimize the requirement for evaluating hundreds of thousands of candidate compounds virtually [10, 11]. In instances where protein structures haven’t been reported, or the potential binding pockets are not determined, 1D and 2D methods can significantly enhance the ligand-based generation approaches. This is particularly beneficial if there are available hit molecules. These techniques effectively design molecules based on their ligand characteristics, without explicit information on the protein pocket structure. Moreover, these methods can provide accurate results if they are supported by high-quality assay data. They have proven to be useful for de novo design during later stages of drug design, in addition to their broad use in the early stages. However, 2D generative models have limitations in representing molecular structures and generating chemical diversity. These models are based on linear sequences of atoms and bonds, which restricts their ability to capture structural features such as stereochemistry and conformational flexibility. Additionally, they often rely on predefined templates, leading to the generation of similar molecules with limited novelty. To adequately capture the complexity of the target pocket, more advanced modeling approaches, such as three-dimensional (3D) generative models, are necessary to address these challenges in drug discovery. 3D methods are more tailored for structure-based approaches when the protein structures and binding pockets are already known, which can generate de novo molecules with explicit three-dimensional coordinates based on the information of protein pocket structure.

Significant progress has been made in the field of 3D generative models, including variational autoencoders (VAE) [12, 13], convolution neural network (CNN) [14], generative adversarial network (GAN) [15, 16], and graph neural network (GNN) [17]. These advances have enabled the development of deep learning models capable of directly generating de novo molecules in the 3D space. Among these approaches, Pocket2Mol has emerged as a noteworthy innovation, enhancing both efficiency and molecule quality compared to previous structure-based drug design models [18]. More specifically, Pocket2Mol is a novel E(3)-equivariant generative neural network that has been pretrained on the CrossDock dataset. It effectively captures spatial and bonding relationships between atoms within the binding pockets. The conditional molecular sampling algorithm employed by Pocket2Mol demonstrates efficiency in characterizing novel position generation strategies and accurately predicting element types without relying on MCMC (Markov chain Monte Carlo) [19]. Importantly, molecules sampled from Pocket2Mol exhibit significantly improved binding affinity as validated through experimental evaluations [18].

Antagonism of protein–protein interactions (PPIs) with small molecules is increasingly considered as a viable therapeutic strategy [20, 21]. Successful PPI inhibitors tend to target proteins that possess deep partner-binding pockets rather than the flat protein interacting surfaces. The WD40 repeat (WDR) domain-containing proteins comprise one of the most abundant PPI domains in the human proteome, playing crucial roles in various cellular processes, including numerous disease-associated mechanisms [22, 23]. Despite lacking clinical validation, WDR5, a novel target with cautious optimism for the treatment of leukemia and other cancer types, has garnered significant attention [24]. Multiple efforts have been undertaken to discover binders for the two distinct peptide-binding pockets present on this scaffold protein, the WIN (WDR5-interacting) pocket and the WBM (WDR5-binding motif) pocket [25]. The proto-oncogene MYC interacts with WDR5 on the WBM interface [26, 27], making it a great drug target for employing a 3D generative modeling approach. The aim is to enable the structural diversity of hits, thereby expanding drug discovery efforts for MYC through this co-factor [28].

In this study, we developed a hit identification pipeline, i.e. Pocket Crafter, that leverages a 3D generative chemistry method to generate novel active molecules as early hits. We utilized this pipeline to propose hit molecules for WDR5 in the 3D space, specifically targeting its WBM pocket that interacts with the oncogenic factor MYC. To evaluate the potential hits, we conducted in vitro biological assays on WDR5 and identified a novel chemical series exhibiting clustered activity. This chemical series demonstrated the ability to disrupt WDR5-MYC interaction in the biochemical assay and acted as binders to WDR5 in the biophysical assay. Together with this case study, our work represents an end-to-end 3D generative chemistry workflow as a viable approach for discovering novel active compounds in the early drug discovery phase.

## Materials and methods

### Pocket Crafter workflow overview

**Fig. 1:**
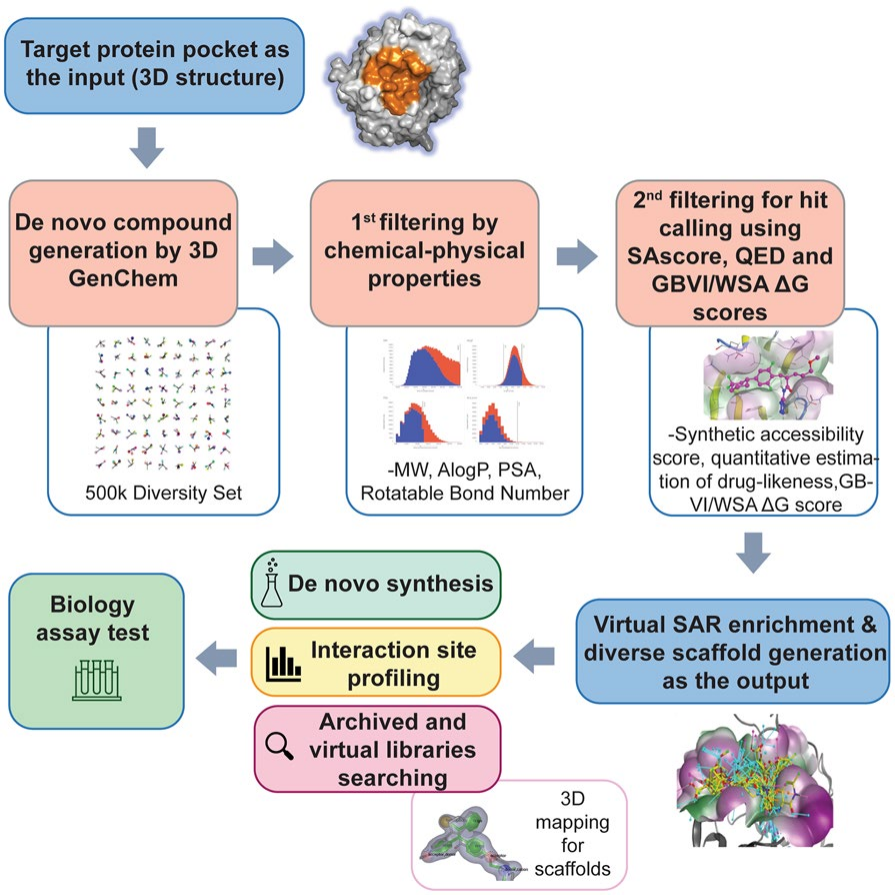
Workflow of Pocket Crafter. The integrated modules and data flow are illustrated. The overall process is shown as following: tertiary protein pocket structure as the input; de novo binder generation with Pocket2Mol 3D generative chemistry approach; chemical-physical property filters; hit calling filters; virtual hit chemotype enrichment (SAR enrichment); the output of Pocket Crafter are novel diverse hit scaffolds with binding pose in tertiary protein pocket; interaction site profiling or de novo synthesis or archived library searching could be followed to generate a focused set of compounds for biological test

The Pocket Crafter workflow has been developed to construct de novo compounds in 3D by crafting atoms and bonds that precisely fit into specific tertiary protein pockets. Unlike methods dependent on reference ligands, our workflow enables thorough exploration of the desired pocket's characteristics. Virtually, it generates small molecule concepts for a wide range of chemical structures and identifies candidate hit compounds through chemotype enrichment, providing guidance to drug discovery projects not only for novel chemical synthesis but also for biological profiling of the interaction site. The overall Pocket Crafter workflow is depicted in Fig. 1. It is a two-step scalable automated workflow suitable for GPU/CPU high-performance computing and cloud environments, which sample code and example dataset is available in supplementary additional file 1.

### Input of protein pocket information for diverse hit generation

Pocket Crafter workflow starts from a pre-defined tertiary protein structure with the 3D coordinates of the binding pocket centroid. The workflow requires the protein or pocket tertiary structure information as the input, typically provided as a set of files, such as PDB (Protein Data Bank) files. To prepare the protein pocket tertiary structure, the Molecular Operating Environment (MOE) QuickPrep module was employed using default settings [29]. Subsequently, the 3D generative chemistry algorithm, Pocket2Mol, was integrated into the workflow with the goal of generating a large and diverse set of virtual 3D molecules that fit the binding pocket [18]. Pocket2Mol utilizes a graph-based approach, sequentially adding one atom/bond at a time based on learned relationships.

In this workflow for our case study, we initially utilized the Pocket2Mol code available on GitHub [18], then increased the sampling parameters to 2000 and repeated the process 300 times with different random seeds to obtain a diverse set of molecular candidates. Consequently, over 500 thousand de novo compounds were generated for the protein pocket, showcasing enhanced diversity within the generated molecules.

### Primary filtering for chemical-physical properties

**Fig. 2:**
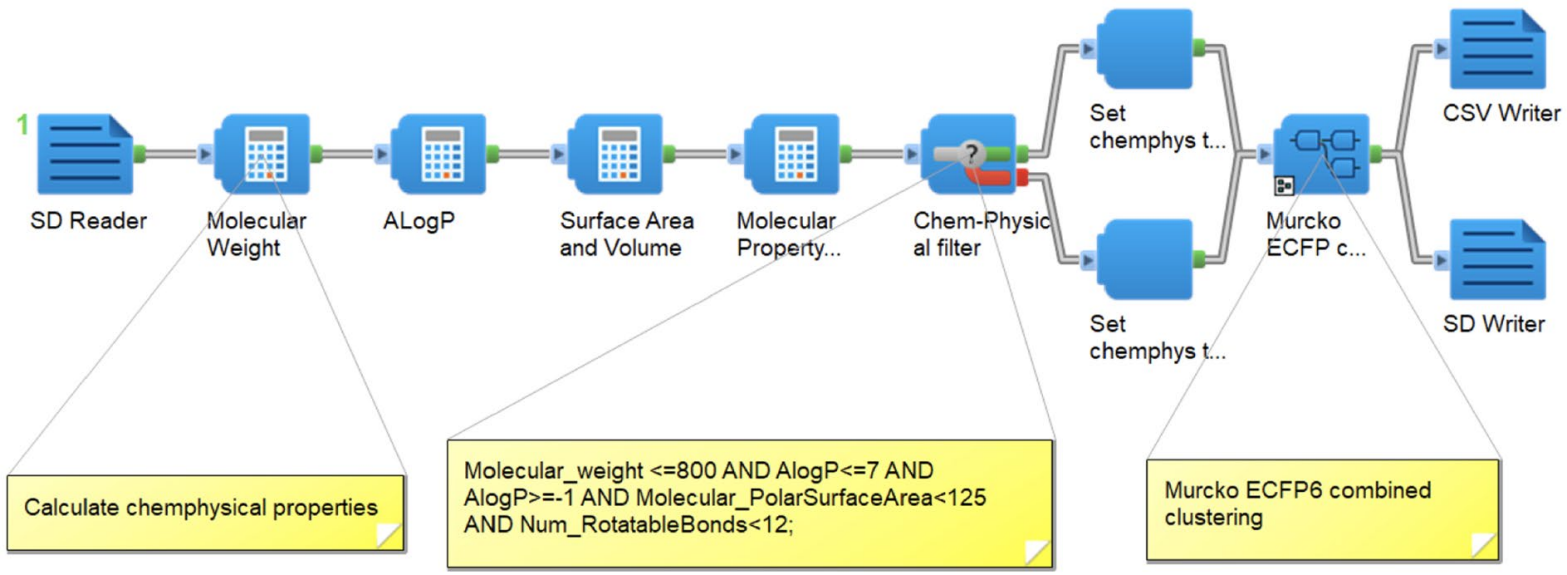
Pipeline Pilot protocol for chemical-physical properties filtering and "Bemis-Murcko Assemblies" clustering. Components in Pipeline Pilot protocol and the parameter cutoffs are illustrated here and also in supplementary additional file 2. Chemical-physical property calculation including molecular weight (Molecular_weight), AlogP, molecular polar surface area (Molecular_PolarSurfaceArea or PSA), and number of rotatable bonds (Num_RotatableBonds)

After generating half a million compounds in SDF (structure data files) format, we employed Pipeline Pilot [30] to validate the generated molecules (Fig. 2), ensuring the correctness of atom type, valency, and charge. Subsequently, we performed calculations for chemical-physical properties, which included molecular weight (Molecular_weight), AlogP, molecular polar surface area (Molecular_PolarSurfaceArea), and number of rotatable bonds (Num_RotatableBonds). Next, we introduced a new Boolean field and incorporated it into the molecule SDF file as a filter for chemical-physical properties. The filter is assigned a value of “true” if the following criteria are satisfied: Molecular_weight is no greater than 800, AlogP is between -1 and 7, Molecular_PolarSurfaceArea (Å^2^) is less than 125, and Num_RotatableBonds is less than 12. Otherwise, the filter is set to “false”.

### Secondary filtering for hit calling using SAscore, QED and GBVI/WSA ΔG scores

Following the initial filtering, firstly all compounds that passed the chemical-physical properties filters were subjected to further filtering using the synthetic accessibility score (SAscore or SAS) [31] and quantitative estimation of drug-likeness (QED) score [32, 33] obtained from RDKit with default parameter settings. In our approximation, the SAscore is calculated as a combination of two components [31]:

1 $$\mathrm{SA score }=\mathrm{ Fragment Score}-\mathrm{ Complexity Penalty}$$

The QED measurement relies on empirical reasoning, which considers the distribution of various molecular properties. These properties include molecular weight, LogP (partition coefficient), topological polar surface area, the count of hydrogen bond donors and acceptors, the number of aromatic rings and rotatable bonds, as well as the presence of undesired chemical functionalities. By taking into account these diverse factors, the QED measurement offers a comprehensive evaluation of molecular quality and desirability as potential drug candidates [34]. The complete weighted QED equation from the original development [32], where *W* represents the weighting for each respective desirability function, is as follows:

2 $$QE{D}_{w}={\text{exp}}\left[\frac{\begin{array}{c}{W}_{MW}{\text{ln}}{d}_{MW}+{W}_{ALOGP}{\text{ln}}{d}_{ALOGP}+{W}_{HBA}{\text{ln}}{d}_{HBA}+{W}_{HBD}{\text{ln}}{d}_{HBD}\\ +{W}_{PSA}{\text{ln}}{d}_{PSA}+{W}_{ROTB}{\text{ln}}{d}_{ROTB}+{W}_{AROM}{\text{ln}}{d}_{AROM}+{W}_{ALERTS}{\text{ln}}{d}_{ALERTS}\end{array}}{{W}_{MV}+{W}_{ALOGP}+{W}_{HBA}+{W}_{HBD}+{W}_{PSA}+{W}_{ROTB}+{W}_{AROM}+{W}_{ALERTS}}\right]$$

Subsequently, two new Boolean fields, namely SAS and QED filters, are generated and included in the molecule SDF file. The SAS filter is assigned a value of “true” if the SAscore is ≤ 4, otherwise it is set to “false”. The QED filter is set to "true" if the QED score is ≥ 0.5, otherwise it is set to “false”.

Then we employed the structure-based clustering to group the generated compounds. Using the aforementioned Pipeline Pilot, we fragmented and annotated all the molecules with Murcko fragment SMILES using the “Bemis-Murcko Assemblies” fragmentation approach [35]. Bemis-Murcko assemblies define a ring system and any chain that connects two or more rings, while removing other chains from the molecule. Subsequently, the molecules were clustered based on ECFP_6 descriptors of Murcko SMILES using the maximum dissimilarity method for selecting cluster centers and a maximum distance of 0.625. Finally, all compounds were annotated with a cluster ID (identification) and the number of compounds in each cluster (Fig. 2).

Secondly, for all the molecules that passed the chemical-physical property filters, we calculated the GBVI/WSA ΔG score as the predicted binding affinity for each compound-protein pair in MOE [29], which script can be found in supplementary additional file 3. The GBVI/WSA ΔG score is a forcefield-based scoring function that estimates the free energy of binding for the ligand in a given pose. It has been trained using the MMFF94x and AMBER99 forcefields on a training set of 99 protein–ligand complexes from the SIE dataset [36]. The functional form of the GBVI/WSA ΔG score is a sum of terms:

3 $$\Delta G\approx c+\alpha \left[\frac{2}{3}\left(\Delta {E}_{{\text{Coul}}}+\Delta {E}_{{\text{sol}}}\right)+\Delta {E}_{{\text{vdW}}}+\beta\Delta S{A}_{{\text{weighted}}}\right]$$

where:

*c* represents the average gain/loss of rotational and translational entropy.

*α, β*are constants which were determined during training (along with *c* and are forcefield-dependent. If not using an AMBER forcefield, the parameters will be set by default to the MMFF trained parameters.

*E*_*Coul*_is the coulombic electrostatic term which is calculated using currently loaded charges, using a constant dielectric of *εi* = 1.

*E*_*sol*_ is the solvation electrostatic term which is calculated using the GB/VI solvation model.

*E*_*vdW*_is the van der Waals contribution to binding.

*SA*_*weighted*_ is the surface area, weighted by exposure. This weighting scheme penalizes exposed surface area.

For the calculation of GBVI/WSA ΔG score in MOE, we utilized the Docking module with the input of the protein pocket’s tertiary structure. If the original tertiary structure contains a co-crystallized ligand in the protein pocket, that molecule is used in the Docking workflow after being prepared with the QuickPrep module using the default settings. However, if the original tertiary structure is an apo protein structure, dummy atoms (LP atoms with no bonded neighbors) were employed in the Docking workflow to designate the pocket location. In the Docking workflow, since our focus is the GBVI/WSA ΔG score calculation rather than the actual docking, we maintained all default parameters except for specific docking parameters: we utilized the existing ligand conformation if available, selected “None” for the placement method, and chose “Rigid Receptor” with a termination criterion of 0.1 gradient for receptor refinement.

### Virtual hit chemotype enrichment (SAR enrichment) and diverse scaffold generation

After completing the two filtering steps, the dataset is now ready for virtual SAR enrichment analysis. The concept is to utilize the statistical Fisher test p-value to determine which hit scaffolds is more abundant in the de novo compounds generated from the 3D generative chemistry model. This information can then guide us to focus on these enriched hit scaffolds for subsequent library screening. Firstly, the molecules that passed the SAscore, QED score, and GBVI/WSA ΔG score filters were defined as virtual hits. Taking into account the variation in binding pockets across different protein families, the workflow facilitated incremental exploration of virtual SAR enrichment analysis by employing a range of GBVI/WSA ΔG score cutoffs, starting from GBVI/WSA ΔG score of -6 and incrementing in intervals of -0.1. This ensured the generation of 100 to 200 top diverse hit SAR scaffolds.

To determine if there was a statistically significant association between the assigned cluster ID (SAR) and the hit calling annotation within the cluster, we performed Fisher’s exact test. Fisher’s exact test is a statistical significance test developed by Ronald Fisher, a renowned statistician [37–39]. It is widely used for the analysis of contingency tables, particularly for small sample sizes, but is also applicable to datasets of all sizes. Fisher's exact test falls under the category of exact tests, as it calculates the exact significance of the deviation from a null hypothesis, providing a precise *p*-value. This characteristic distinguishes Fisher's exact test from other tests that rely on approximations, which are accurate only when the sample size approaches infinity. The equation for Fisher's exact test is as follows:

4 $$p=\frac{\left(a+b\right)!\left(c+d\right)!\left(a+c\right)!\left(b+d\right)!}{a!b!c!d!n!}$$

where:

*p* = *p-value*

a, b, c, d = values in a contingency table

*n* = *total frequency*

The GBVI/WSA ΔG score represents the potential energy change that occurs when the protein and ligand interact. A higher negative score indicates a stronger binding affinity, while a lower negative or positive score suggests the weaker or non-existent binding. In this context, we consider de novo compounds with a GBVI/WSA ΔG score of -6 or lower as virtual hits, indicating a higher likelihood of being true binders that should be further validated through experimental verification. In our workflow, for any molecule in the test, it can only be assigned as “yes” or “no” for the hit, and “yes” or “no’ for a cluster ID. *a*, *b*, *c*, *d* are the structure counts in the contingency table for hit vs.(versus) not-hit and in-this-cluster vs. not-in-this-cluster, and *n* is the total number of the structures. After the test, any cluster showing *p*-value less than or equal to 0.05 is considered as a hit enriched SAR cluster. This virtual SAR enrichment analysis allows us to identify which chemical groups and structures are most likely to be the true binders for the pocket with dynamic SAR range suitable for medicinal chemists to further optimize. We then selected the top GBVI/WSA ΔG score molecule(s) from each cluster as the hit scaffold(s). By selecting the top compound(s) from each hit cluster, we were able to focus on the most promising binders for the next step processing: as the starting point for de novo synthesis and further potency and property improvement, or the archived and virtual libraries searching, as well as the protein ligand interaction site profiling.

### Archived and virtual libraries searching

To create molecules that meet pharmaceutical standards, including desired biological activity, target selectivity, and drug properties relevant to pharmacokinetics and pharmacodynamics, challenges often arise due to the synthesis of these proposed molecules. Beside this, the de novo synthesis of the novel scaffolds generated by generative chemistry algorithms might be challenging due to the inherent complexity of these virtual molecules. A portion of the generated molecules may have intricate and unconventional architectures or possess unique chemical features and functional groups that are not commonly found in existing compounds. The synthesis may involve multiple steps or requires the use of specialized reagents or reaction conditions that may not be readily available. This lack of precedent might make it difficult to develop efficient synthetic strategies to access these novel structures. The complexity, coupled with limitations in scalability and cost-efficiency, poses practical hurdles for the experimental synthesis, even when considering only a subset of these molecules [40, 41]. To address this, we propose an intermediate approach that involves conducting searches in archived and virtual libraries. This approach can be seamlessly integrated with virtual screening techniques and subsequently validated through wet lab experiments, such as high throughput biological assays. This approach involves utilizing existing small molecule libraries, either commercial or those available within the research unit. In our case, we utilized a Novartis internal diverse library containing 3 to 4 million compounds, as well as an external Enamine REAL database with coverage of 4 to 10 billion compounds that can be synthesized on-demand [42]. Another option is the use of a customized enumeration library, referred to as PFL (Project Focus Library), which can be designed based on reaction schemes and building blocks specific to the target of interest [43].

**Fig. 3:**
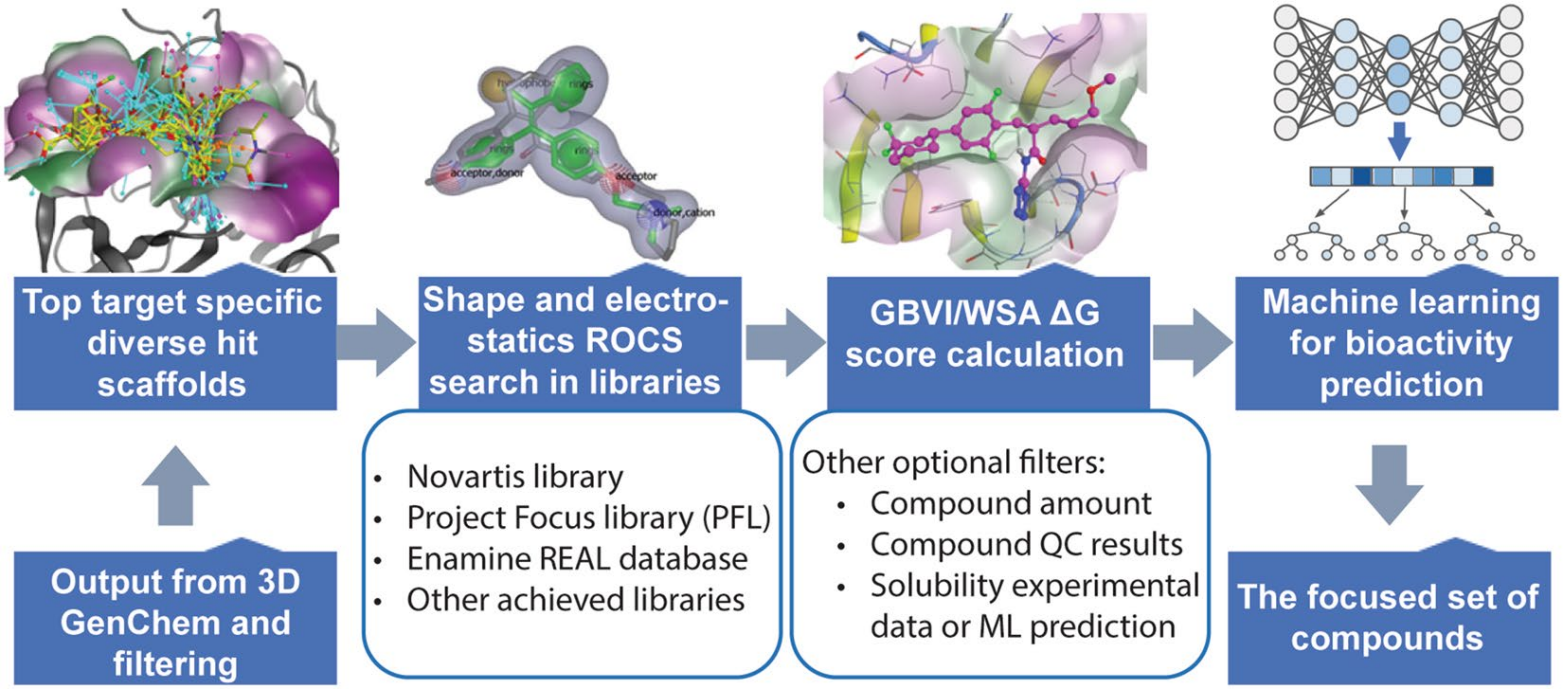
Pipeline for archived and virtual libraries searching. Modules in the pipeline and the parameter cutoffs are illustrated. The process is: diverse hit scaffolds from the output after two layers filtering and SAR enrichment; Shape and electrostatics ROCS searching in libraries; GBVI/WSA ΔG calculation; more optional filters can be added such as compound amount, compound QC results, solubility experimental data or machine learning (ML) prediction, and bioactivity machine learning model prediction, to generate a focused set of compounds for biological testing

Furthermore, once we generated 100–200 top diverse hit scaffolds, as illustrated in Fig. 3, we employed ROCS (Rapid Overlay of Chemical Structures) [44] and other ligand-based machine learning models to search for compounds in the archive or/and prioritize designed compounds synthesis based on shape, electrostatic properties, pharmacophoric features, and other 2D or 3D characteristics of the compounds and protein pocket residues. ROCS is a powerful virtual screening tool known for its ability to rapidly identify putatively active compounds through the shape comparison. It has demonstrated strong competitiveness and often outperforms structure-based approaches in the virtual screening [45–47]. Notably, ROCS has been instrumental in identifying novel and interesting molecular scaffolds, particularly for targets that were traditionally challenging for computational techniques [48]. In our case study, we used individual 3D SDF files of the top 100–200 diverse hit scaffolds as the input for ROCS searching against a pre-compiled Novartis archived library Omega database with a Tanimoto Combo score cutoff of 1.0.

For all the molecules identified from the ROCS results, we calculated the GBVI/WSA ΔG score as the predicted binding affinity for each compound-protein pair using the same MOE method described in detail as above. We applied the same GBVI/WSA ΔG score cutoff as used in the SAR enrichment step. The compounds that passed the GBVI/WSA ΔG score filter underwent further filtering based on (1) available quantity to ensure sufficient amounts for biological testing; (2) LC-QC (Liquid Chromatography-Quantitative Control) filter to ensure matched analytical evaluation of the compounds; (3) Novartis global solubility machine learning prediction and solubility experimental filter to ensure the compounds' solubility for biological testing. Moreover, the filtered compounds eligible for other ligand-based machine learning models can be seamlessly integrated into this workflow for orthogonal screening compound set selection, such as the profile-quantitative structure–activity relationship (pQSAR) models from Novartis [49] or customized models specific to the target's mechanism of action. Ultimately, the selected compound set is subjected to biological activity testing.

The approximate runtime for each protein or pocket calculation is one week using High-Performance Computing (HPC) GPU clusters for Pocket2Mol (10 nodes) and CPU clusters (1–200 nodes).

### Compound QC (quality control) analysis

Purification of the compounds was carried out either using pre-packed silica gel cartridges (Biotage or ISCO) or reverse-phase HPLC (High-performance liquid chromatography) with C18 columns, > 95% purity for all the active compounds tested in the biological assay has been confirmed by analytical HPLC. ^1^H NMR (proton nuclear magnetic resonance) spectra were recorded in acetonitrile-*d*_3_ or methanol-*d*_4_, on Bruker NMR spectrometer with 400 or 500 MHz 1H Larmor frequency. NMR chemical shifts (δ) were quoted in parts per million (ppm) and are reported relative to residual nondeuterated solvent signals. Coupling constants are reported in Hertz (Hz). Splitting patterns are indicated as follows: br, broad; s, singlet; d, doublet; t, triplet; q, quartet; dd, doublet of doublets, m, multiples.

### WDR5 biochemical HTRF (homogeneous time resolved fluorescence) assay

The assay was adapted [50] and performed in 384-well white OptiPlate plate (PerkinElmer) for compound single dose (40 μM) or the dose response assay. A mixture of protein and peptide was added into the well and incubated for 20 min. Compounds in DMSO were dispensed at a 14-point, 3.16-fold dilution scheme with the top concentration of 75 μM. A mixture of HTRF detection antibodies was then added and incubated for 1 h before plate reading (Perkin Elmer, Envision). The final assay component concentrations are 6 nM WDR5 (N-His, 1–334), 50 nM MYC MbIIIb peptide (256–268 a.a., QEDEEEIDVVSVE-GKK-Biotin-OH), 1.5 nM Eu-anti-His-Ab (PerkinElmer, AD0401) and 3 µg mL-1 Sreptavidin-Surelight APC (PerkinElmer, AD0201) in the assay buffer of 25 mM HEPES (pH = 7.5), 0.05% v/v Tween-20, 100 mM NaCl, 2 mM DTT and 0.1% w/v BSA. For the counter assay, protein pair of WDR5 and MYC was replaced with a biotin-PEG-PEG-6xHis peptide (GenScript), to evaluate if the compound interferes with elements of the assay format. This helps to characterize the specific activity of the compound against the PPI of WDR5 and MYC in HTRF. Dose response curves and half-maximal inhibitory concentration (IC50) values were generated by GraphPad Prism. The representative curves are based on the mean values from at least two independent experiments in triplicates.

### WDR5 differential scanning fluorimetry (DSF) assay

The compound was dissolved in DMSO at a concentration of 10 mM before mixing with WDR5. 2 µM full-length WDR5 protein was mixed with 5 × SYPRO Orange (Thermo Fisher Scientific, S6650) in DSF assay buffer (20 mM HEPES pH = 8.0, 150 mM NaCl) and then mixed with 200 µM compound in a 384-well PCR plate. The mixture solution was then incubated with shaking for 5 min before running the thermal melting experiments on the CFX384 Touch™ Real-Time PCR detection system (Bio-Rad). The samples were heated from 20 to 95 °C at a rate of 0.5 °C/30 s. The melting curve and peak data was analyzed by a modified Boltzmann equation using a Novartis in-house program. The reported Tm (melting temperature) values are based on the mean values from two independent experiments in triplicates.

## Results and discussion

**Fig. 4:**
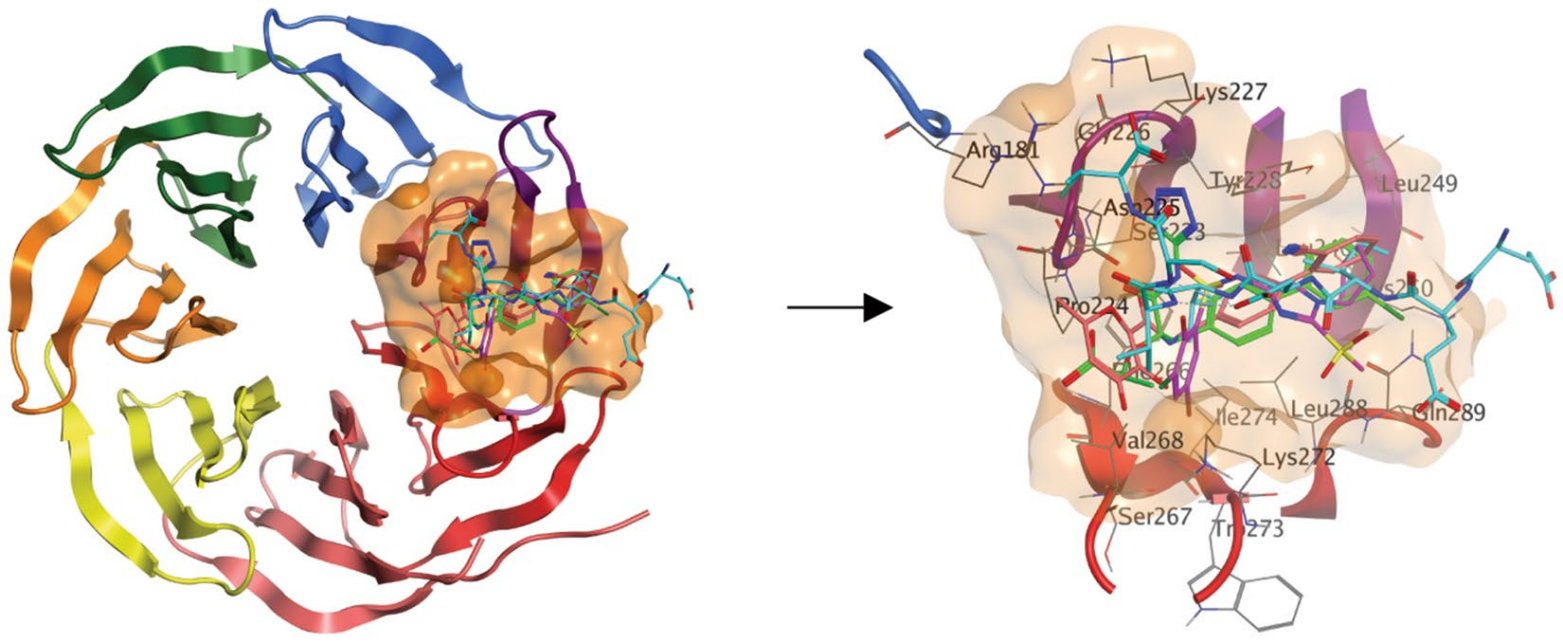
Overview of the WDR5 WBM pocket and the known binding molecules. Left Panel: WBM pocket (orange) on WDR5 protein (β-propeller blades each in a different color). Right Panel: zoom-in of WBM pocket showing ligands with binding mode illustration. Ligand structures were superimposed with MOE. MYC MbIIIb peptide (Cyan, PDB: 4Y7R), WM-662 (Green, PDB: 8F1G) and Compound 1 (Red, PDB: 8G3C) from Novartis, and Compound 12 from the Fesik lab (Magenta, PDB: 6UOZ)

The interaction between WDR5 WBM pocket (highlighted by orange color in Fig. 4) and MYC is involved in MYC’s association with chromatin and required for its oncogenic function in cancers [26, 27]. Disrupting the WDR5-MYC interaction might be a promising approach for targeting MYC through WDR5, as a few WBM pocket binders have been identified and published by the Fesik lab and us [50–53]. How MYC peptide and these known small molecules bind to WBM pocket is presented by the zoom-in diagram in the right panel of Fig. 4. These small molecules or their corresponding original hits were acquired through wet lab experimental activities such as fragment-based screening or high throughput biochemical screening. Taken together, the biological significance of targeting this WBM pocket in MYC or WDR5 related diseases, the knowledge of existing screening results and the feasibility of using the validated binders as the benchmark really makes it a good case study to validate the Pocket Crafter workflow that we elucidated in Fig. 1–3.

### Diverse scaffold generation through Pocket Crafter

In order to explore the chemical space beyond the existing chemical matters for the WDR5 WBM pocket, we employed the Pocket Crafter workflow (depicted in Fig. 1). This innovative methodology allows for the generation of molecular entities tailored to complement the unique 3D topology of specific protein pockets. To achieve this, we utilized 3D generative chemistry model with the known tertiary structure of WDR5 (PDB: 8F1G) [50], and successfully generated an expansive library consisting of 543,491 distinct and valid structures, thereby significantly expanding the diversity of chemical entities available for investigation in this context.

**Fig.5:**
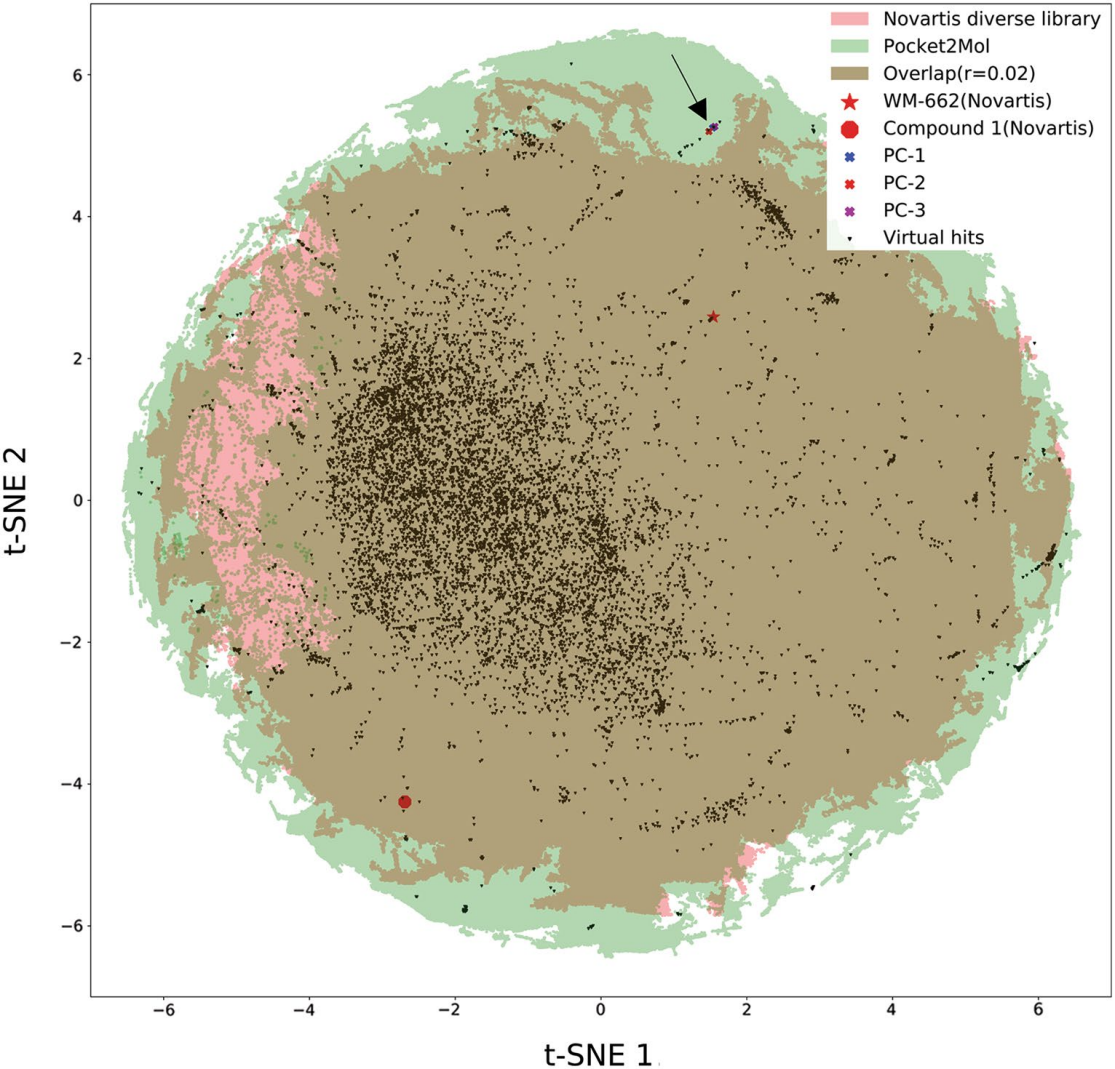
Chemical space map representation for compounds generated by Pocket Crafter or the reported HTRF screening. t-SNE was used for visualizing data by giving each datapoint a location in this two-dimensional map. The diversity of chemical space generated from Pocket2Mol is represented in green. In comparison, compounds obtained from Novartis diverse library HTRF experimental screening are depicted in pink. The overlapped chemical space (based on a buffered radius of 0.02) between Pocket2Mol and Novartis HTRF screened library is represented by the light brown. The two published WDR5 WBM binder scaffolds from HTRF screening, namely WM-662 (indicated by a red star) and Compound 1 (depicted by a red circle), are among the virtual hits generated by the workflow after filtering and hit calling (shown in dark brown). The three novel hits from Pocket Crafter, PC-1, PC-2 and PC-3 (related to Fig. 9) are marked by cross symbols in blue, red and purple respectively, which are not covered by HTRF screen with Novartis library

A 2-dimensional chemical space map was constructed using two datasets (Fig. 5): a total of 543,491 virtual compound set (represented in green) was generated via Pocket Crafter workflow; a total of 1,101,793 compound set was screened at Novartis (represented in pink) to identify inhibitors of WDR5-MYC PPI using biochemical HTRF. Combining these datasets, this map was created using the R programming language, utilizing chemical-physical properties and ECFP-6 descriptors, with the assistance of the Rtsne function, t-distributed stochastic neighbor embedding (or t-SNE), a statistical method for visualizing high-dimensional data by giving each datapoint a location in a two or three-dimensional map. In order to illustrate the chemical space coverage connection between the two sets (Pocket2Mol and Novartis HTRF screen library) visually, we applied buffering to each data point in the t-SNE space as above, and then created circles with a fixed radius of r = 0.02. Subsequently, the circles were combined for both the Pocket2Mol (green) and Novartis HTRF (pink) sets. The light brown region represented the overlap of the chemical space as a function with radius of 0.02 in Fig. 5. This chemical space map effectively demonstrates a broader coverage of the chemical landscape from Pocket2Mol, especially by the green “edge” area of the data point collection, highlighting the diversity of chemical scaffolds over Novartis library, even though this is an extremely diversified library by designing [54].

### Postprocessing results with two layers of filters

**Fig.6:**
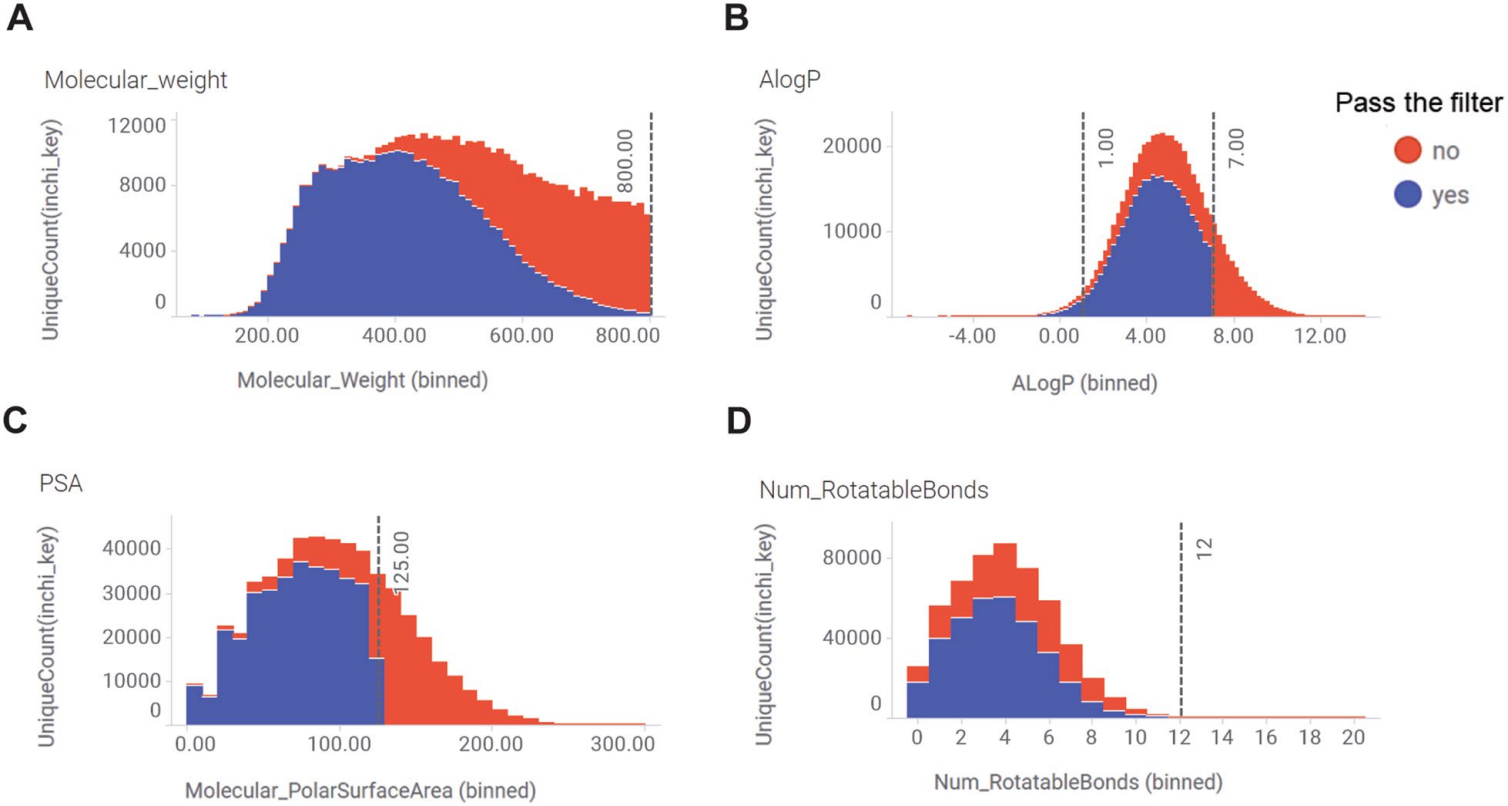
Histograms of WDR5 compounds distribution from Pocket Crafter after chemical-physical properties filtering. Filters are: **A** Molecular Weight (Molecular_weight) ≤ 800; **B** AlogP between -1 and 7; **C** Molecular Polar Surface Area (Molecular_PolarSurfaceArea or PSA) (Å^2^) < 125; **D** Number of Rotatable Bonds (Num_RotatableBonds) < 12. In each histogram, blue means compounds that passed all the other three filters and red means the compounds that failed either of the other three filters

After the calculations to determine the chemical-physical properties of the generated compounds and data visualization in Fig. 6, we applied a set of filters based on specific criteria (molecular weight, AlogP, Molecular Polar Surface Area, and Number of Rotatable Bonds) as depicted in Fig. 6A–D. As a result, 352,820 structures successfully met the selection criteria and advanced to the subsequent stage of analysis and evaluation.

**Fig.7:**
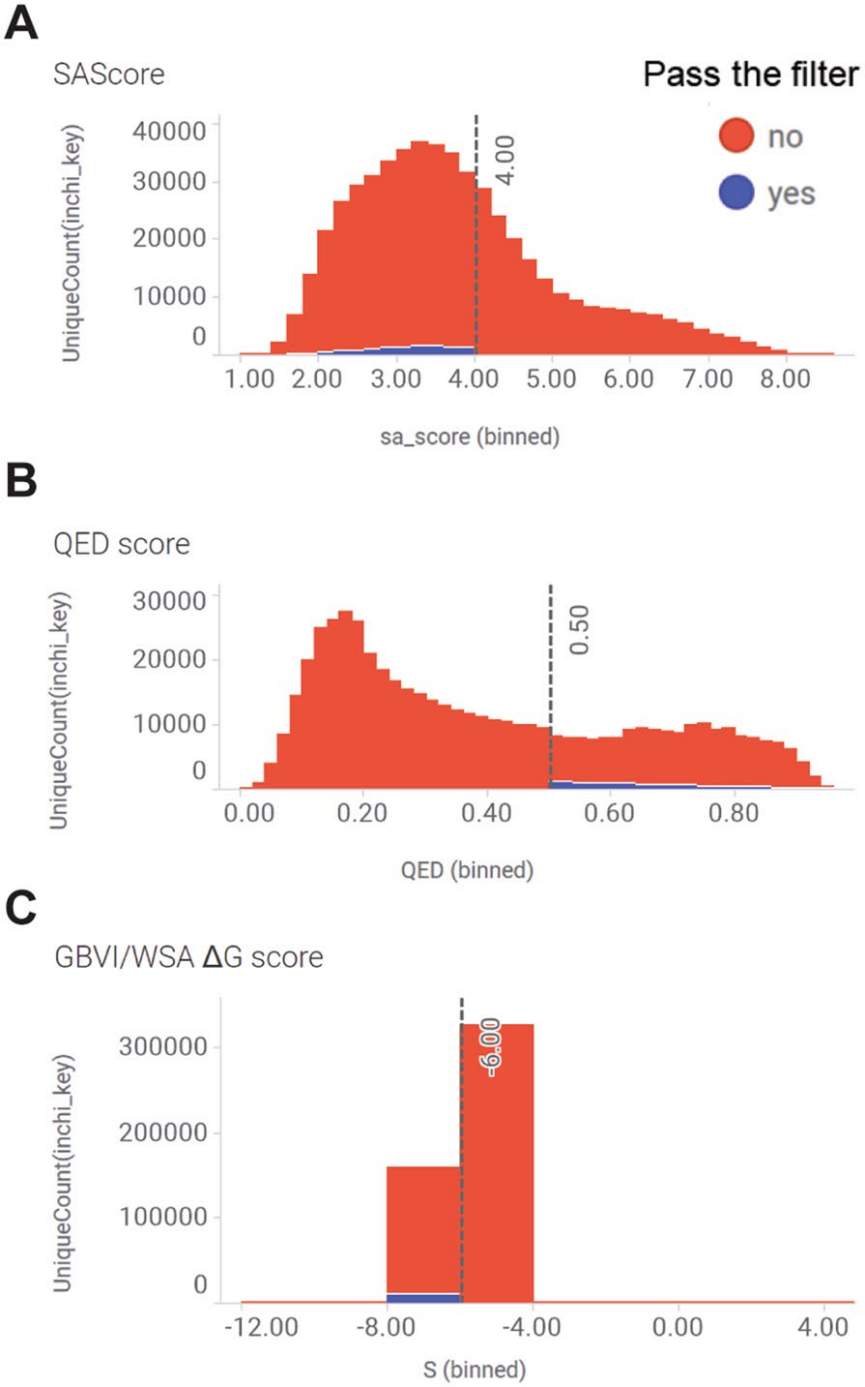
Histograms of WDR5 compounds distribution after 2nd layer filtering for hit calling. Molecules that passed the chemical physical-property filters are further calculated for **A** SAScore, **B** QED score and **C** GBVI/WSA ΔG score with the cutoffs in Table 1. In each histogram, blue means compounds that passed both the other two hit calling filters, and red means the compounds that failed either of the other two filters

352,820 novel virtual structures pose a significant challenge for early drug discovery, particularly concerning follow-up activities such as synthesis. This challenge is commonly encountered in generative chemistry. While generative chemistry garners increasing attention, the critical task of ensuring the synthetic feasibility of the generated molecules remains paramount. Bridging the gap between the innovative potential of generative chemistry and its practical application in synthesis is crucial for successful integration into drug discovery endeavors. To overcome this challenge, we conducted a comprehensive analysis on the 352,820 molecules that passed the chemical-physical property filters. These molecules were further evaluated based on their SAscore, QED score and GBVI/WSA ΔG score, employing specific cutoff values outlined in Table 1. The distribution of compounds by these hit calling parameters was thoroughly examined and visualized in Fig. 7A–D.

**Table 1:** Secondary layer filters for WDR5 hit calling in Pocket Crafter workflow

Filter	Criteria cutoff
SAscore	≤ 4
QED score	≥ 0.5
GBVI/WSA ΔG score	≤ -6

Following this subsequent hit calling filtering process, we successfully identified 9531 virtual hits that exhibited favorable properties. These hits were then subjected to in silico SAR enrichment analysis, enabling a more profound exploration of their potential as promising candidates in the field of drug discovery. As we mentioned that Novartis library used for HTRF screen covers about 1.1 million druglike diverse compounds, so it is not surprising that many virtual hits from Pocket Crafter overlap with these structures in Novartis library, just showing as those dark brown dots in the light brown area of Fig. 5. In this figure, it also reveals an intriguing coincidence: two hit scaffolds, WM-662 [50] and Compound 1 [53], previously identified from the experimental HTRF screen and published by us, are highlighted in red and aligned with the virtual hits identified through the Pocket Crafter workflow. This “overlapped hit” observation proves the great potential of this workflow to generate hit compounds virtually with high relevance to the target, as the pocket binders. More encouragingly, this workflow can go beyond our diverse library and generate novel “hits”, as many dark brown dots are outside of the overlapped area, which means they are distinct structures not covered by the 1.1 million diverse compound collection.

The Pocket Crafter and traditional virtual docking approaches can both be utilized for virtual screening of our internal compound library, which has approximately 3 million compounds. The Pocket Crafter approach employs a generative algorithm to construct compounds atom-by-atom and bond-by-bond within a 3D binding pocket, effectively exploring the pocket's chemical space. Subsequently, diverse top virtual hit scaffolds are used to map compounds onto the binding pocket via ROCS. This method is highly efficient and suitable for ultra-large virtual screening (ULVS). Also confirmed hits from Pocket Crafter tend to be more drug-like, chemically diverse, and target selective compared to those from virtual docking. Despite both approaches being applicable to structure-based virtual screening, the compound lists generated by Pocket Crafter and virtual docking have limited overlap due to significant differences in their algorithms and workflows.

### SAR enrichment analysis and archived and virtual libraries searching

To enhance our analysis, we developed a separate comprehensive compound library searching pipeline, as illustrated in Fig. 3, that leverages essential information obtained from the potential hit compounds. This pipeline incorporates the extraction of critical features, including shape, electrostatic properties, and pharmacophoric characteristics. By focusing on these key attributes, we were able to narrow down the selection of compounds for subsequent library screening, significantly improving the efficiency and success rate of the hit discovery process. The selected compounds can be ordered directly from existing small molecule libraries or prioritized for synthesis, then tested.

In this WDR5 case study, we utilized Novartis internal archived diverse library consisting of 3 to 4 million compounds. Beyond this, multiple compound sources can be utilized potentially in this pipeline including an external Enamine REAL database covering 4 to 10 billion compounds and a customized enumeration library. For all the molecules obtained from the ROCS analysis, we calculated the GBVI/WSA ΔG score and further refined the compound selection based on availability, solubility, and quality control results. This rigorous selection process resulted in a focused set of 2029 compounds for subsequent biological testing.

### Hit confirmation and data comparison with Novartis diverse library HTRF screening results

**Fig.8:**
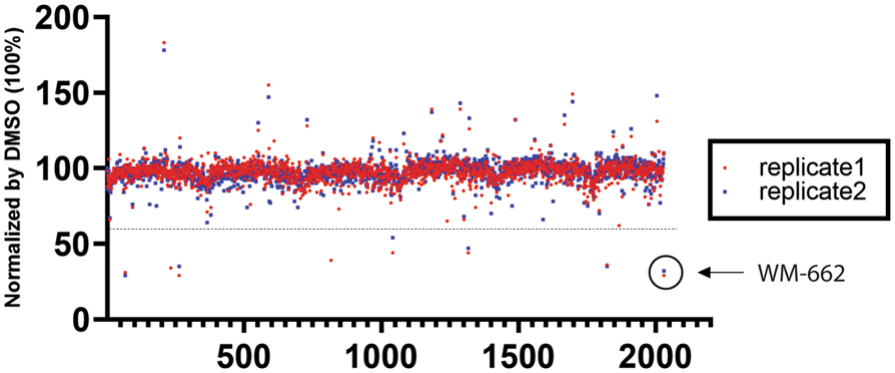
Distribution of HTRF activities for 2029 compounds. 2029 compounds were tested in biochemical WDR5-MYC PPI HTRF assay at 40 μM with duplicates, each indicated in red or blue. The control DMSO activity was set up as 100% for normalization. Compounds showing greater than 40% inhibition were considered as the primary hits. The published probe WM-662 was highlighted by the circle as the positive control inhibitor. X-axis: compound. Y-axis: HTRF activity. Dash line: 40% inhibition

We tested this 2029 compound set by 40 μM dose in WDR5-MYC PPI biochemical HTRF assay, with the similar assay condition used in the previous biochemical screening (Novartis diverse library HTRF screen) which led to the finding of WM-662 and Compound 1 [50, 53], the known WBM pocket binding molecules. The compound activity distribution was presented in a dot plot (Fig. 8) that shows 7 compounds inhibited the assay signal greater than 40%, which was the same criteria we applied to select primary hits in the early experimental HTRF screen using the library of around 1.1 million compounds. WM-662 showing 70% inhibition was used as the positive control. Notably, our efforts leveraging Pocket Crafter on WDR5 have yielded encouraging outcome for the hit rate. Through the implementation of this integrated workflow, we have achieved a substantial 12.8-fold increase in the hit rate compared to the early diversity library HTRF screening of 1,101,793 compounds (Table 2). This significant improvement highlights the effectiveness of our tailored compound generation strategy in enhancing the chance of identifying compounds as primary hits against the target protein.

**Table 2:** Hit summary and comparison with Novartis diverse library HTRF screen results

Hit generation approach	Compound number	Hit Number (cutoff: 40% inhibition at 40 μM)	Hit Rate
Novartis diverse library HTRF screen	1,101,793	2715	0.025%
Pocket Crafter	2029	7	0.345%

### Biological activity profiling for WDR5 hit compounds

**Fig.9:**
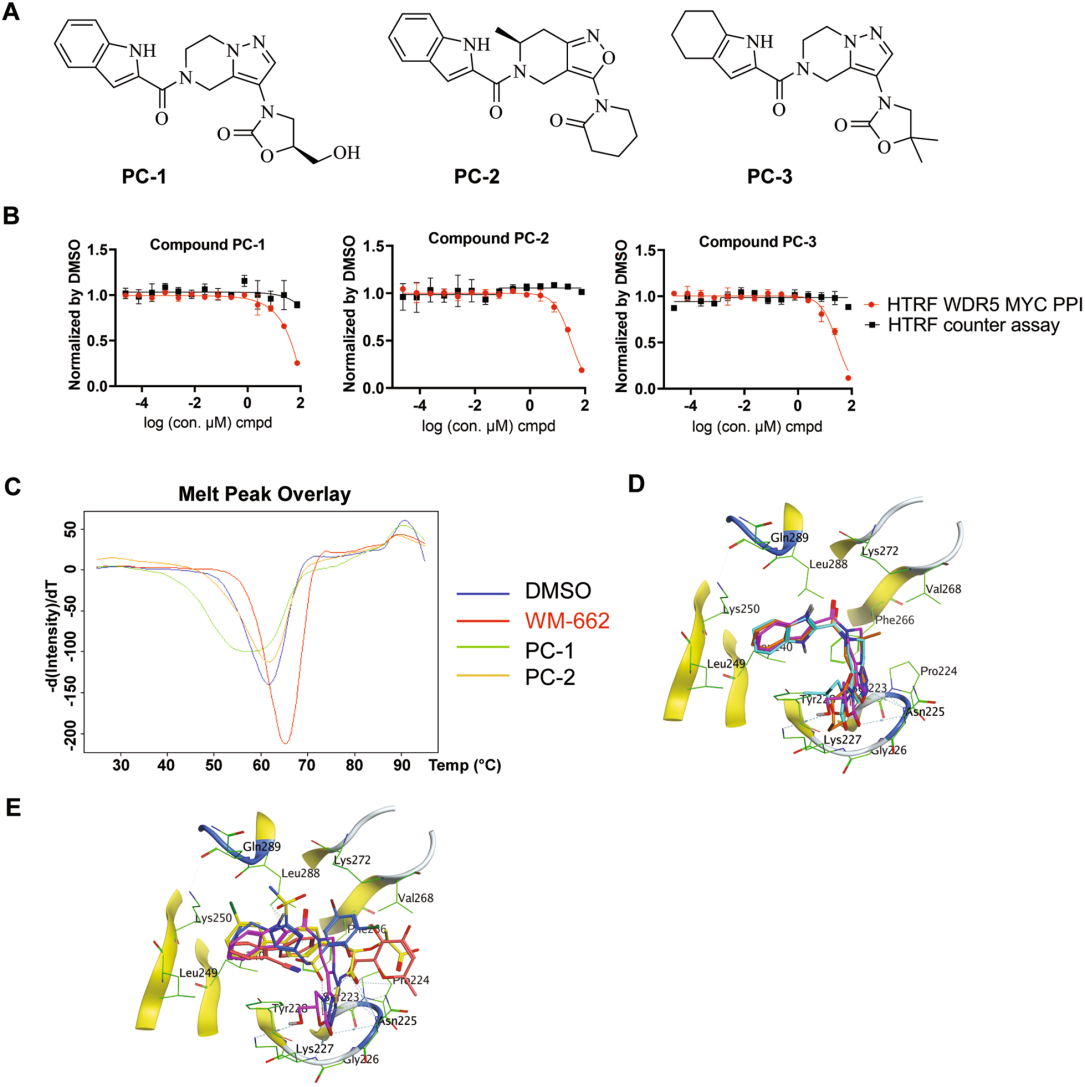
Biological activity validation for WDR5 hit compounds from Pocket Crafter.** A** Chemical structures of the three compounds showing IC50 < 50 μM in HTRF assay, named PC-1, PC-2 and PC-3, **B** Dose response curves fitted from HTRF assay. IC50 = 35.6 ± 0.6, 27.5 ± 3.9 and 28.5 ± 0.2 μM respectively for compounds PC-1, PC-2, and PC-3 in the binding assay and all IC50s > 75 μM in the counter assay, **C** Melting peaks in WDR5 DSF by compound treatment. Thermal (Tm) shift: -4.2 °C by PC-1 and − 1.2 °C by PC-2 compared to the DMSO control. WM-662 was tested together as an assay control showing Tm shift of 3.2 °C, **D** Binding modes proposed by Pocket Crafter for the three hit compounds in WDR5 (yellow ribbon). PC-1 (Magenta), PC-2 (Orange) and PC3 (Cyan), **E** The comparison of binding modes among PC-1 (Magenta), WM-662 (Yellow), Compound 1 (red) and the Fesik lab’s Compound 12 (blue)

Next, we further conducted WDR5-MYC HTRF dose–response curve (DRC) study to analyze the single dose activity from the primary hits more quantitatively. Three out of the 7 primary hits (Fig. 9A) showed good dose response curve fitting and the IC50s of 35.6, 27.5 and 28.5 μM respectively for compound PC-1, PC-2 and PC-3 (Fig. 9B), reaffirming the in vitro potency of these hit compounds biochemically from single dose testing. Even though their activities are a little weaker than WM-662 or Compound 1 (18 or 14 μM) in this WDR5-MYC HTRF assay [46, 49], they don’t hit the HTRF counter assay as the flat black curves indicated in Fig. 9B, suggesting the true specificity of these compounds in disrupting WDR5 and MYC interaction.

Moreover, we tested the binding of these hit compounds to WDR5 in an orthogonal biophysical assay. The direct binding of PC-1 and PC-2 to WDR5 was demonstrated by Differential Scanning Fluorimetry (DSF) assay (Fig. 9C). Thermal shift analysis showed that adding PC-1 or PC-2 to WDR5 resulted in a negative Tm (melting temperature) shift of 4.2 °C or 1.2 °C in compound treated groups, compared to DMSO control. WM-662 was tested together as a binder control with the validated shift, which triggered the Tm shift of 3.2 °C. This Tm shift in DSF indicates that the PC-1 or PC-2 compound can work on WDR5 protein as the true binder from this biophysical readout. The negative Tm shift indicates that there might be an interaction between the compound and the protein that is destabilizing to the protein's tertiary structure [55], since the positive or negative Tm shift is dependent on the compound’s preference for binding either the native state of the protein or a less populated conformational state, such as a partially unfolded state that is energetically close to the native state. Basically, the compound influences the conformational equilibrium and determines the direction of the Tm shift.

We mapped these three hits back to the data points in Fig. 5, as the arrow indicated in the upper right corner. They are in the green area, not covered by HTRF screened Novartis library, which supports again this workflow can identify new binder chemotype. Interestingly, in addition to the aggregation pattern they showed up in the map, we did notice these two compounds and the biochemically active compound PC-3 show certain structure and binding mode similarity (Fig. 9A and D), suggesting Structure–Activity Relationship empirically for further medicinal chemistry exploration. They also showed different binding mode to known WDR5 binding small molecules such as WM-662, Compound 1 or the Fesik lab’s Compound 12 putatively, from the superimposed results of X-ray co-structures and the binding visualization by Pocket Crafter (Fig. 9E), suggesting they are indeed novel hits. The quality analysis results for these three archived compounds are as below:

Compound PC-1: LC–MS: *m/z* = 382.1 [M + H]^+^. ^1^H NMR (500 MHz, acetonitrile-*d*_3_) δ = 9.90 (s, 1H), 7.71 (d, *J* = 8.1 Hz, 1H), 7.62–7.43 (m, 2H), 7.30 (ddd, *J* = 8.2, 7.0, 1.2 Hz, 1H), 7.15 (ddd, *J* = 8.0, 7.0, 1.0 Hz, 1H), 6.98 (d, *J* = 2.2 Hz, 1H), 5.09 (s, 2H), 4.70 (dq, *J* = 9.3, 4.0 Hz, 1H), 4.28 (s, 4H), 3.96 (t, *J* = 8.8 Hz, 1H), 3.86–3.61 (m, 3H).

Compound PC-2: LC–MS: *m/z* = 379.8 [M + H]^+^0.1H NMR (500 MHz, acetonitrile-*d*_3_) δ = 9.82 (s, 1H), 7.69 (d, *J* = 8.0 Hz, 1H), 7.52 (d, *J* = 8.3 Hz, 1H), 7.29 (t, *J* = 7.7 Hz, 1H), 7.14 (q, *J* = 7.5 Hz, 1H), 6.91 (d, *J* = 2.2 Hz, 1H), 5.48–5.17 (m, 2H), 4.28 (s, 1H), 3.82 (tt, *J* = 11.7, 6.2 Hz, 2H), 3.08 (d, *J* = 16.6 Hz, 1H), 2.79 (d, *J* = 16.3 Hz, 2H), 2.53 (t, *J* = 6.4 Hz, 4H), 1.97 (dt, *J* = 5.0, 2.5 Hz, 4H), 1.28 (d, *J* = 7.0 Hz, 3H).

Compound PC-3: LC–MS: *m/z* = 384.3 [M + H]^+^.^1^H NMR (400 MHz, methanol-*d*_4_) δ = 7.57 (s, 1H), 6.45 (d, *J* = 2.3 Hz, 1H), 5.02 (s, 2H), 4.24 (q, *J* = 3.4 Hz, 4H), 3.77 (s, 2H), 2.60 (t, *J* = 6.1 Hz, 2H), 2.52 (t, *J* = 6.0 Hz, 2H), 1.85—1.71 (m, 4H), 1.54 (s, 6H).

### Protein–ligand interaction profiling for other novel scaffolds

Our study offers an intriguing application wherein we identify new hit scaffolds that deviate from the existing SAR scaffolds explored on the same target. The discovery of these novel scaffolds presents exciting prospects for chemical optimization and the exploration of unexplored regions on the protein surface within the pocket.

**Fig. 10:**
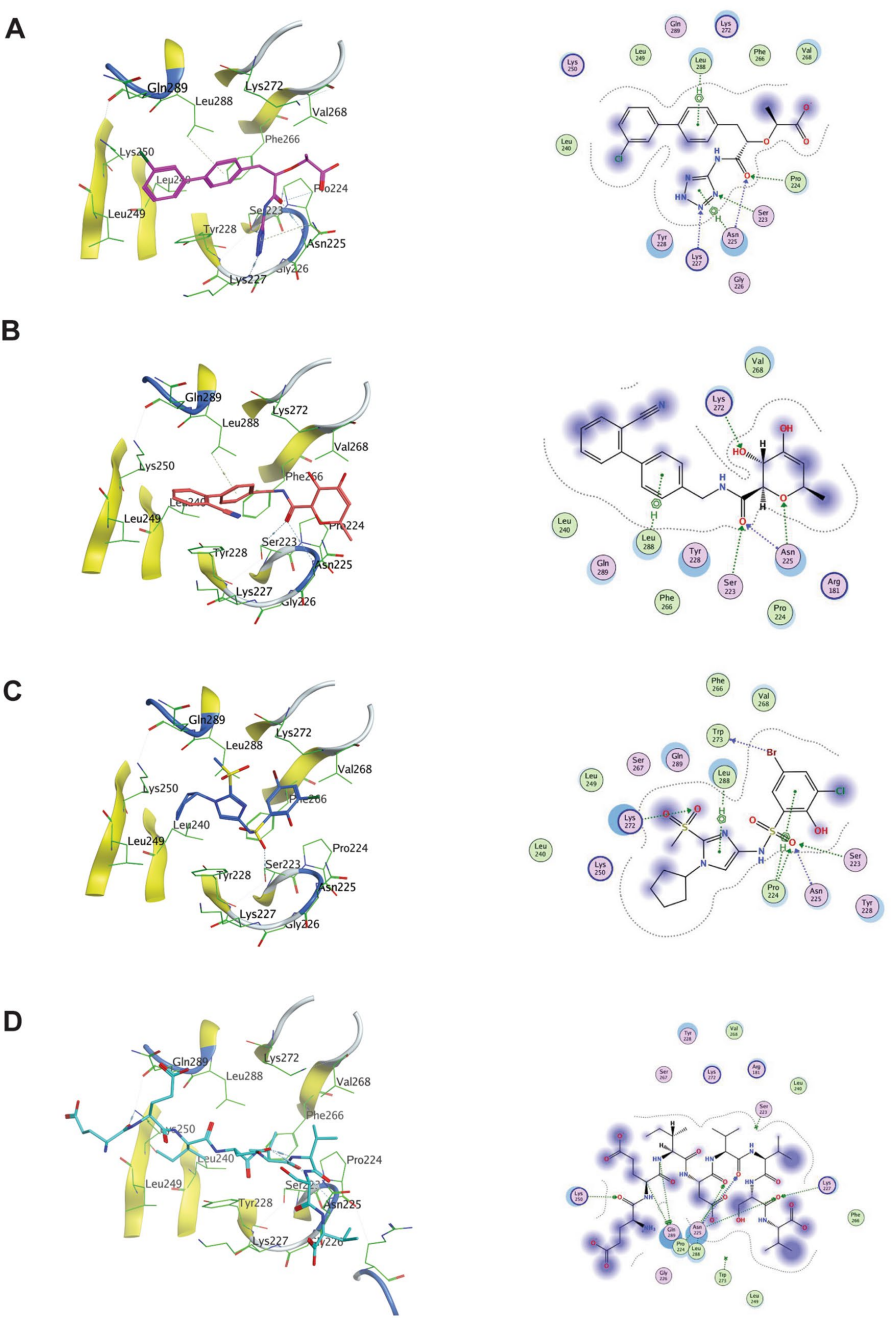
Binding modes of different WBM binders to WDR5. Individual binding mode visualization of WBM pocket binders and protein–ligand interaction diagram for **A** WM-662, PDB: 8F1G, **B** compound 1, PDB: 8G3C, **C** compound 12 from the Fesik lab, PDB: 6UOZ, **D** MYC MbIIIb peptide, PDB: 4Y7R

To assess the effectiveness of our approach, we conducted protein–ligand interaction profiling, comparing the interactions of known scaffolds or the MYC MbIIIb peptide (Fig. 10A–D) with those hits generated through the Pocket Crafter workflow. Remarkably, our approach successfully generated molecules that exhibited similar interaction patterns to those observed in X-ray co-crystal structures. These generated molecules effectively engaged in all the key interactions with Ser223, Pro224, Asn225, Lys227, Lys272, and Leu288 on the WDR5 protein.

**Fig. 11:**
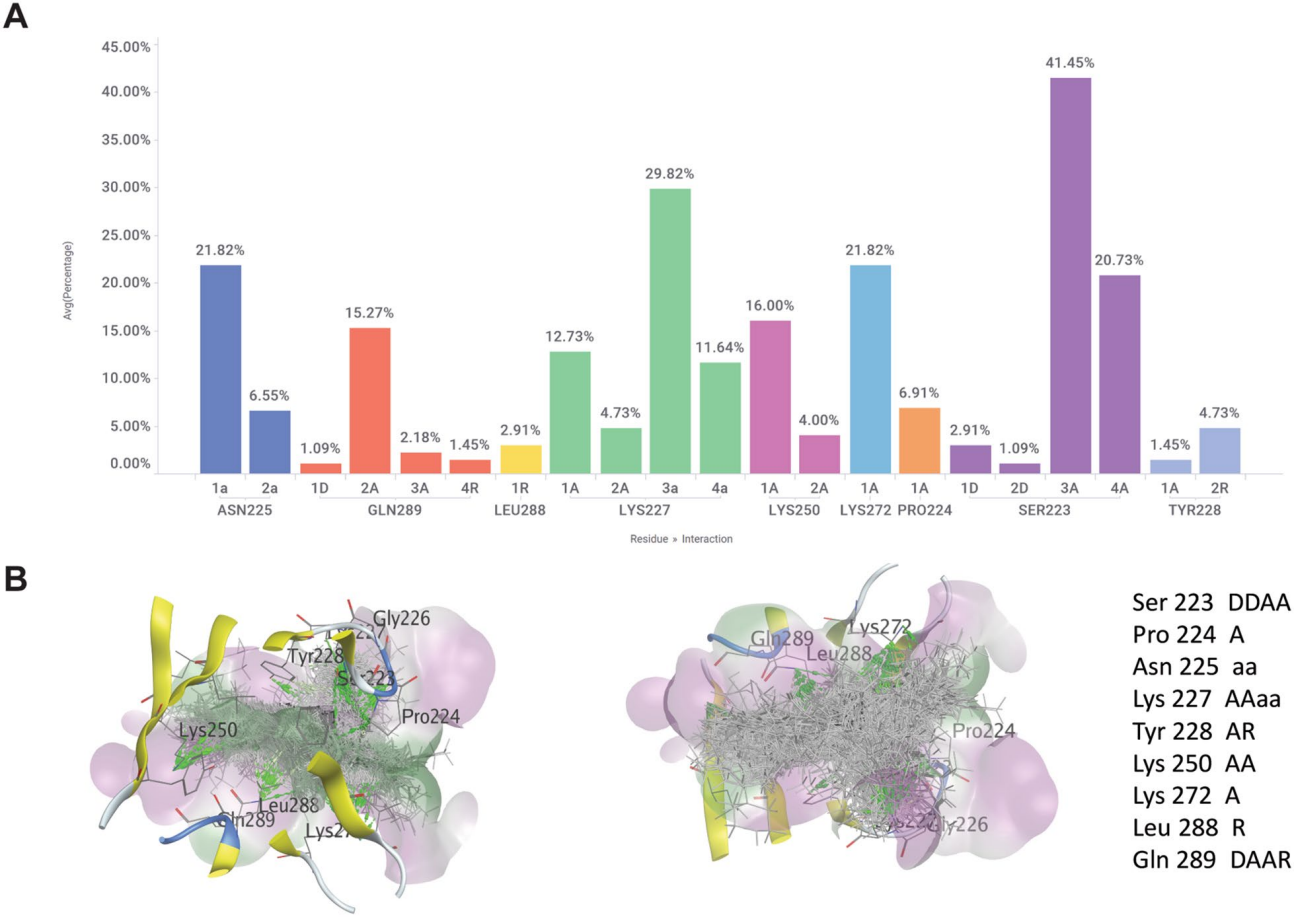
Protein–ligand interaction profiling with the hits generated from Pocket Crafter. **A** Protein–ligand interaction profiling results from MOE PLIF histogram showing the number of Pocket Crafter diverse hit scaffolds (relative frequency indicated by Y-axis, or the bar height) with each amino acid residue plotted in the X-axis. Each fingerprint bit is denoted by a character to indicate its meaning. D sidechain hydrogen bond donor. A: sidechain hydrogen bond acceptor. a: backbone hydrogen bond acceptor. R: arene attraction. **B** Protein ligand hydrogen bond interaction map (highlighted in green) between WDR5 (PDB: 8F1G) and top diverse hit scaffolds selected from initial 3D generative chemistry compounds

Furthermore, the protein–ligand interaction profile generated from the top diverse chemotypes produced by our workflow facilitated the identification of all the key WDR5 residues involved in interacting with the MYC MbIIIb peptide (Fig. 11A). This includes not only the residues that interact with known ligands (Pro224, Asn225, Lys227, and Lys888) but also additional residues (Lys250 and Glu289) that were previously unexplored in the hit molecules. These newly identified residues hold significant potential for the design of compounds that can effectively engage them as the stronger WDR5 binder, particularly in the context of competition with MYC MbIIIb peptide, for instance the optimization of WM-662 to WM-586 that can engage Lys250 significantly improved the potency in the SAR study of that scaffold series [50]. Detailed interactions between the key residues and the top diverse hits selected from Pocket Crafter workflow are shown in Fig. 11A and the Additional file 1: Table S1 the Protein Ligand Interaction Fingerprints (PLIF) summary. This interaction histogram is generated with the residue-ligand interaction abundance percentage data from the PLIF tool in MOE using WDR5 tertiary structure (PDB: 8F1G) and top hit scaffolds. It summarizes the interactions between ligands and proteins using a fingerprint scheme. Interactions such as hydrogen bonds, ionic interactions and surface contacts are classified according to the residue of origin and built into a fingerprint scheme which is representative of a given database of protein–ligand complexes. Furthermore, it illustrates 3D view of all the top diverse hits in the pocket of WDR5 with hydrogen bond interactions in Fig. 11B (shown in green) with the key residues identified in Fig. 11A.

Moreover, the protein–ligand interaction profile revealed another key WDR5 residue, Tyr 228, which interacts with diverse scaffolds generated through the Pocket Crafter workflow. This finding underscores the versatility of our approach in generating compounds that interact with diverse regions and key residues, thereby expanding the chemical space and offering new avenues for further exploration and optimization.

## Conclusions

We have developed the Pocket Crafter workflow to carry out virtual hit identification in early drug discovery using a 3D generative chemistry approach. As a case study, the Pocket Crafter workflow has facilitated the hit identification for the WDR5 WBM pocket successfully, with a higher hit rate compared with the experimental HTRF screening results. The biological activity of these hits has been validated through in vitro assays, confirming their role as genuine WDR5 binders and disruptors of the WDR5-MYC interaction. The discovery of this new chemical series marks a promising starting point for WDR5 drug development. Through the efficient exploration of a vast chemical space and the incorporation of pertinent structural information, we have showcased the significant potential and efficacy of this workflow in expediting the identification of potential drug candidates. This approach opens up new avenues for the development of innovative therapies to address unmet medical needs, offering expanded possibilities in the field of drug discovery.

### Supplementary Information

**Additional file 1: **Pocket Crafter workflow process sample code and example dataset (ChEMBL). SMILES Molecular formula strings. **Table S1.** Protein Ligand Interaction Fingerprints (PLIF) summary. **Table S2.** Novartis diverse library HTRF screen result dataset. **Table S3.** ChEMBL compound set screen HTRF result. **Figure S1.** Hit CHEMBL1081548 activity confirmation in WDR5 HTRF.

**Additional file 2. **Pipeline Pilot protocol for chemical-physical properties filtering and clustering (xml)

**Additional file 3. **MOE GBVI/WSA ΔG calculation shell script (sh).

## Data Availability

Pocket2Mol, its source code and models are freely available from Github (https://github.com/pengxingang/Pocket2Mol). The MOE, OpenEye, R and Pipeline Pilot program packages are commercial software with paid licenses. The RDkit library including SAscore and QED score codes are free of charge in RDKit, and its components are mostly released under the BSD 2-Clause License. Sample calculations and our scripts for Pocket Crafter workflow are available in the supporting information as it showed in sample code and example dataset session using ChEMBL open database. The 1.1 million compounds HTRF screen dataset used for comparison with Pocket Crafter results in this study was attached in supporting information Additional file 1: Table S2. 2029 compounds HTRF data was shown in Fig. 8. Example set of ChEMBL compound screen HTRF result was shown in Additional file 1: Table S3 and Figure S1. Molecular structures are shown in the figures and SMILEs are in supporting information. All X-ray structure data are available in PDB public data sources as the IDs indicated.

## Abbreviations

3D: Three-dimensional
SMILES: Simplified molecular-input line-entry system
PPI: Protein–protein interactions
WBM: WDR5-binding motif
WIN: WDR5-interacting site
PDB: Protein data bank
SAR: Structure–activity relationship
MOE: Molecular operating environment
QED: Quantitative estimation of drug-likeness
ROCS: Rapid overlay of chemical structures
QC: Quality control
HTRF: Homogeneous time resolved fluorescence
IC50: Half-maximal inhibitory concentration
DSF: Differential scanning fluorimetry

## References

[1] Hughes J, Rees S, Kalindjian S, Philpott K (2011). Principles of early drug discovery. Br J Pharmacol.

[2] Vamathevan J, Clark D, Czodrowski P (2019). Applications of machine learning in drug discovery and development. Nat Rev Drug Discov.

[3] Gupta R, Srivastava D, Sahu M (2021). Artificial intelligence to deep learning: machine intelligence approach for drug discovery. Mol Divers.

[4] Batool M, Ahmad B, Choi S (2019). A structure-based drug discovery paradigm. Int J Mol Sci.

[5] Sanchez-Lengeling B (1979). Aspuru-Guzik A (2018) Inverse molecular design using machine learning: generative models for matter engineering. Science.

[6] Winter R, Montanari F, Steffen A (2019). Efficient multi-objective molecular optimization in a continuous latent space. Chem Sci.

[7] Arús-Pous J, Johansson SV, Prykhodko O (2019). Randomized SMILES strings improve the quality of molecular generative models. J Cheminform.

[8] Bjerrum EJ (2017) SMILES Enumeration as data augmentation for neural network modeling of molecules. arXiv:170307076. 10.48550/arXiv170307076

[9] Li Y, Zhang L, Liu Z (2018). Multi-objective *de novo* drug design with conditional graph generative model. J Cheminform.

[10] Bort W, Baskin II, Gimadiev T (2021). Discovery of novel chemical reactions by deep generative recurrent neural network. Sci Rep.

[11] Zhavoronkov A, Ivanenkov YA, Aliper A (2019). Deep learning enables rapid identification of potent DDR1 kinase inhibitors. Nat Biotechnol.

[12] Blaschke T, Olivecrona M, Engkvist O (2018). Application of generative autoencoder in De Novo molecular design. Mol Inform.

[13] Gómez-Bombarelli R, Wei JN, Duvenaud D (2018). Automatic chemical design using a data-driven continuous representation of molecules. ACS Cent Sci.

[14] Valueva MV, Nagornov NN, Lyakhov PA (2020). Application of the residue number system to reduce hardware costs of the convolutional neural network implementation. Math Comput Simul.

[15] Sanchez-Lengeling B, Outeiral C, Guimaraes GL, Aspuru-Guzik A (2017). An objective-reinforced generative adversarial network for inverse-design chemistry (ORGANIC). ChemRxiv.

[16] Prykhodko O, Johansson SV, Kotsias P-C (2019). A *de novo* molecular generation method using latent vector based generative adversarial network. J Cheminform.

[17] Kipf TN, Welling M (2016) Semi-Supervised Classification with Graph Convolutional Networks. arXiv: 160902907. 10. 48550/arXiv160902907

[18] Peng X, Luo S, Guan J, et al (2022) Pocket2Mol: Efficient Molecular Sampling Based on 3D Protein Pockets. In the 39th International Conference on Machine Learning, Proceedings of Machine Learning Research, 162. https://proceedings.mlr.press/v162/peng22b.html, pp 17644–17655

[19] Gamerman D, Lopes HF (2006). Markov chain Monte Carlo: stochastic simulation for bayesian inference.

[20] Arkin MR, Tang Y, Wells JA (2014). Small-molecule inhibitors of protein-protein interactions: progressing toward the reality. Chem Biol.

[21] Mabonga L, Kappo AP (2019). Protein-protein interaction modulators: advances, successes and remaining challenges. Biophys Rev.

[22] Xu C, Min J (2011). Structure and function of WD40 domain proteins. Protein Cell.

[23] Schapira M, Tyers M, Torrent M, Arrowsmith CH (2017). WD40 repeat domain proteins: a novel target class?. Nat Rev Drug Discov.

[24] Guarnaccia A, Tansey W (2018). Moonlighting with WDR5: a cellular multitasker. J Clin Med.

[25] Chen X, Xu J, Wang X (2021). Targeting WD repeat-containing protein 5 (WDR5): a medicinal chemistry perspective. J Med Chem.

[26] Thomas LR, Wang Q, Grieb BC (2015). Interaction with WDR5 promotes target gene recognition and tumorigenesis by MYC. Mol Cell.

[27] Thomas LR, Adams CM, Wang J (2019). Interaction of the oncoprotein transcription factor MYC with its chromatin cofactor WDR5 is essential for tumor maintenance. Proc Natl Acad Sci.

[28] Mullard A (2022). Climbing cancer’s MYC mountain. Nat Rev Drug Discov.

[29] Molecular Operating Environment release 2022.02 (2023). Chemical computing group ULC; Montreal, QC, Canada. https://www.chemcomp.com/index.htm

[30] Pipeline Pilot release 2020 (2023). BIOVIA, dassault systèmes, San Diego. https://www.3ds.com/products-services/biovia/products/data-science/pipeline-pilot/

[31] Ertl P, Schuffenhauer A (2009). Estimation of synthetic accessibility score of drug-like molecules based on molecular complexity and fragment contributions. J Cheminform.

[32] Bickerton GR, Paolini GV, Besnard J (2012). Quantifying the chemical beauty of drugs. Nat Chem.

[33] Wildman SA, Crippen GM (1999). Prediction of Physicochemical Parameters by Atomic Contributions. J Chem Inf Comput Sci.

[34] Landrum G (2023) QED module in RDKit: Open-source cheminformatics software. http://www.rdkit.org. Accessed 1 Mar 2023.

[35] Bemis GW, Murcko MA (1996). The properties of known drugs. 1 Molecular frameworks. J Med Chem.

[36] Naïm M, Bhat S, Rankin KN (2007). Solvated interaction energy (SIE) for scoring protein−ligand binding affinities. 1. Exploring the parameter space. J Chem Inf Model.

[37] Fisher RA (1922). On the interpretation of χ2 from contingency tables, and the calculation of P. J Roy Stat Soc.

[38] Fisher RA (1954). Statistical methods for research workers.

[39] Agresti A (1992). A survey of exact inference for contingency tables. Stat Sci.

[40] Segler MHS, Preuss M, Waller MP (2018). Planning chemical syntheses with deep neural networks and symbolic AI. Nature.

[41] Jiménez-Luna J, Grisoni F, Schneider G (2020). Drug discovery with explainable artificial intelligence. Nat Mach Intell.

[42] Shivanyuk A, Ryabukhin S, Bogolyubsky A (2007). Enamine REAL database: making chemical diversity real. Chem Today.

[43] Saldívar-González FI, Huerta-García CS, Medina-Franco JL (2020). Chemoinformatics-based enumeration of chemical libraries: a tutorial. J Cheminform.

[44] ROCS v3.5.1.2 (2022), OpenEye scientific software, Santa Fe, NM. http://www.eyesopen.com

[45] Hawkins PCD, Skillman AG, Nicholls A (2007). Comparison of shape-matching and docking as virtual screening tools. J Med Chem.

[46] Venhorst J, Núñez S, Terpstra JW, Kruse CG (2008). Assessment of Scaffold hopping efficiency by use of molecular interaction fingerprints. J Med Chem.

[47] Sheridan RP, McGaughey GB, Cornell WD (2008). Multiple protein structures and multiple ligands: effects on the apparent goodness of virtual screening results. J Comput Aided Mol Des.

[48] Rush TS, Grant JA, Mosyak L, Nicholls A (2005). A shape-based 3-D Scaffold hopping method and its application to a bacterial protein−protein interaction. J Med Chem.

[49] Martin EJ, Polyakov VR, Zhu X-W (2019). All-assay-Max2 pQSAR: activity predictions as accurate as four-concentration IC _50_ s for 8558 Novartis assays. J Chem Inf Model.

[50] Ding J, Li G, Liu H (2023). Discovery of potent small-molecule inhibitors of WDR5-MYC interaction. ACS Chem Biol.

[51] Macdonald JD, Chacón Simon S, Han C (2019). Discovery and optimization of salicylic acid-derived sulfonamide inhibitors of the WD repeat-containing protein 5–MYC protein-protein interaction. J Med Chem.

[52] Chacón Simon S, Wang F, Thomas LR (2020). Discovery of WD repeat-containing protein 5 (WDR5)–myc inhibitors using fragment-based methods and structure-based design. J Med Chem.

[53] Ding J, Liu L, Chiang Y-L (2023). Discovery and structure-based design of inhibitors of the WD repeat-containing protein 5 (WDR5)–MYC interaction. J Med Chem.

[54] Schuffenhauer A, Schneider N, Hintermann S (2020). Evolution of Novartis’ small molecule screening deck design. J Med Chem.

[55] Foulkes DM, Byrne DP, Yeung W (2018). Covalent inhibitors of EGFR family protein kinases induce degradation of human Tribbles 2 (TRIB2) pseudokinase in cancer cells. Sci Signal.

