# Supplementary material for: Pocket Crafter: a 3D generative modeling based workflow for the rapid generation of hit molecules in drug discovery
Source: J Cheminform. 2024 Mar 21;16:33. doi: 10.1186/s13321-024-00829-w (PMC10958880; doi:10.1186/s13321-024-00829-w)

Supplementary information

Pocket Crafter: a 3D generative modeling based workflow for the rapid generation of hit molecules in drug discovery

Lingling Shen[a#*], Jian Fang[a#], Lulu Liu[a], Fei Yang[a], Jeremy L. Jenkins[a], Peter S. Kutchukian[a], He Wang[a*]

[a]Novartis Biomedical Research, Cambridge, MA 02139, USA.

[#]Lingling Shen and Jian Fang contributed equally.

***Correspondence**:

Lingling Shen

lingling.shen@novartis.com

He Wang

he.wang@novartis.com

Supplementary Information includes:

1. Pocket Crafter workflow process sample code and example dataset (ChEMBL)
2. SMILES Molecular formula strings
3. Table S1: Protein Ligand Interaction Fingerprints (PLIF) summary
4. Table S2: Novartis diverse library HTRF screen result dataset
5. Table S3: ChEMBL compound set screen HTRF result
6. Figure S1: Hit CHEMBL1081548 activity confirmation in WDR5 HTRF

1. Sample code and example dataset

For WDR5, we applied the Pocket Crafter workflow with the example dataset (ChEMBL version 20 dataset: https://ftp.ebi.ac.uk/pub/databases/chembl/ChEMBLdb/releases/chembl_20/), and 751 compounds were tested in HTRF. 2 primary hits were obtained with a hit rate of approximately 0.266%, which is comparable with hit rate from Pocket Crafter using the Novartis library (0.345%) and ~10 fold higher than the hit rate from web lab HTRF screen using Novartis diverse library. Compound list, detailed hit activity and confirmation results are shown in Table S3 and Figure S1.

Pocket Crafter workflow process sample code include followings:

1.1 de novo binder generation with Pocket2Mol 3D generative chemistry approach (using P2M)
1.2 Chemical-physical property filters and "Bemis-Murcko Assemblies" clustering. (using Pipeline Pilot)
1.3 Hit calling filters (using SAS)
1.4 Hit calling filters (using QED)
1.5 Archived library searching (using Openeye ROCS)
1.6 Hit calling filters and archived library searching (using MOE GBVI/WSA ΔG calculation)

1.1 Pocket2mol for de novo binder generation:
Download and install Pocket2Mol from https://github.com/pengxingang/Pocket2Mol
Update configs/sample_for_pdb.yml with num_samples: 2000, beam_size: 500, max_steps: 100
In sample_for_pdb.py, replace line 83 with a random seed generator, which is also used as prefix to log_dir at line 86, e.g.

```
run=os.environ["SGE_TASK_ID"]
seed_all(config.sample.seed*int(run))
log_dir = get_new_log_dir(args.outdir, prefix='%s' % (run))
```

Repeat the following sampling 500 times with different seeds: python sample_for_pdb.py --pdb_path xxx.pdb --center " xxxx" --outdir WDR5

1.2 Pipeline Pilot protocol for chemical-physical properties filtering and "Bemis-Murcko Assemblies" clustering:
Install Pipeline Pilot release 2020, and then use the protocol in supplementary file (chemphys-cluster.xml).

1.3 SAS (Synthetic Accessibility Score) for hitcalling:
RDKit-based implementation of SAS is used here with code from github (rdkit/Contrib/SA_Score at master · rdkit/rdkit · GitHub).

1.4 QED (quantitative estimation of drug-likeness) for hicalling:
RDKit-based implementation of QED is used here with code from rdkit QED module. (rdkit.Chem.QED module — The RDKit 2023.09.1 documentation)

1.5 OpenEye ROCS for archived library searching:
Install ROCS v3.5.1.2 from OpenEye 2020, and then use the code as below:

```
DB=/NVS-lib

input=MbIIIb.sdf
query="${input%.*}"

rocs -dbase $DB/*$file.oeb \
    -query ./input/$input \
    -prefix rocs_$query_$file \
    -outputdir ./output  \
    -statusfile processing/rocs_$query_$file.status \
    -logfile processing/rocs_$query_$file.log \
    -oformat sdf \
    -outputquery false \
    -report none \
    -besthits 100000000 \
    -sdTags \
    -cutoff 1.0 \
    -rankby TanimotoCombo
```

1.6 MOE GBVI/WSA ΔG calculation for Hit calling filters and archived library searching: the shell script is attached as moe-dg.sh

2. SMILES Molecular formula strings

Id	Structure
PC-1	OC[C@@H]1CN(C(=O)O1)C2C=NN3CCN(CC=23)C(=O)C5=Cc4ccccc4N5
PC-2	C[C@H]2CC1=NOC(=C1CN2C(=O)C4=Cc3ccccc3N4)N5CCCCC5=O
PC-3	CC1(C)CN(C(=O)O1)C2C=NN3CCN(CC=23)C(=O)C5=CC4CCCCC=4N5

3. Table S1: Protein Ligand Interaction Fingerprints (PLIF) summary

Each fingerprint bit is denoted by a character to indicate its meaning:

**Annotation**	**Description**
-	bit not set
D	sidechain hydrogen bond donor
A	sidechain hydrogen bond acceptor
a	backbone hydrogen bond acceptor
R	arene attraction

PC virtual hits	223 D	223 D	223 A	223 A	224 A	225 a	225 a	227 A	227 A	227 a	227 a	228 A	228 R	250 A	250 A	272 A	288 R	289 D	289 A	289 A	289 R
VH-1	-	-	-	-	-	-	-	-	-	a	-	-	-	-	-	-	-	-	-	-	-
VH-2	-	-	-	-	-	-	-	-	-	a	-	-	-	-	-	-	-	-	-	-	-
VH-3	-	-	-	-	-	-	-	-	-	a	a	-	-	A	-	-	-	-	-	-	-
VH-4	-	-	A	-	-	-	-	-	-	-	-	-	-	-	-	-	-	-	-	-	-
VH-5	-	-	A	-	-	-	-	-	-	-	-	-	-	-	-	-	-	-	-	-	-
VH-6	-	-	A	-	-	a	-	-	-	-	-	-	-	-	-	-	-	-	A	-	-
VH-7	-	-	A	-	-	-	-	-	-	-	-	-	R	-	-	-	-	-	A	A	-
VH-8	-	-	A	A	-	a	a	-	-	-	-	-	-	-	-	A	-	-	A	A	-
VH-9	-	-	-	-	-	-	-	-	-	-	-	-	-	-	-	A	-	-	-	-	-
VH-10	-	-	A	-	-	a	-	-	-	a	a	-	-	-	-	-	-	-	-	-	-
VH-11	-	-	A	A	-	-	-	-	-	-	-	-	R	-	-	-	-	-	-	-	-
VH-12	-	-	-	-	-	-	-	-	-	-	-	-	-	-	-	-	-	-	A	-	-
VH-13	-	-	A	A	-	-	-	A	-	a	a	-	-	A	A	-	-	-	-	-	-
VH-14	-	-	A	A	A	-	-	-	-	-	-	-	-	-	-	-	-	-	-	-	-
VH-15	-	-	-	-	-	-	-	-	-	-	-	-	-	-	-	A	-	-	-	-	-
VH-16	-	-	-	-	-	-	-	A	-	-	-	-	-	-	-	-	-	-	-	-	-
VH-17	-	-	-	-	-	-	-	-	-	-	-	-	-	-	-	-	-	-	A	-	-
VH-18	-	-	-	-	-	-	-	-	-	-	-	-	-	-	-	A	-	-	-	-	-
VH-19	-	-	-	-	A	a	a	-	-	-	-	-	-	-	-	-	-	-	-	-	-
VH-20	-	-	A	A	-	a	-	-	-	-	-	-	-	-	-	-	-	-	-	-	-
VH-21	-	-	-	-	-	-	-	A	-	-	-	-	-	-	-	-	-	-	-	-	-
VH-22	-	-	A	-	-	-	-	A	-	a	a	-	-	-	-	-	-	-	-	-	-
VH-23	-	-	-	-	-	-	-	-	-	-	-	-	-	-	-	-	R	-	-	-	-
VH-24	-	-	-	-	-	-	-	-	-	-	-	-	-	A	-	-	-	-	-	-	-
VH-25	-	-	-	-	A	-	-	-	-	-	-	-	-	-	-	-	-	-	-	-	-
VH-26	-	-	A	A	-	a	-	-	-	-	-	-	-	-	-	-	-	-	-	-	-
VH-27	-	-	A	-	-	-	-	A	-	a	a	-	-	-	-	-	-	-	A	-	-
VH-28	-	-	A	A	-	a	-	-	-	-	-	-	R	-	-	-	-	-	-	-	-
VH-29	-	-	-	-	-	-	-	-	-	-	-	-	-	-	-	A	-	-	-	-	-
VH-30	-	-	-	-	-	-	-	-	-	-	-	-	-	-	-	A	-	-	-	-	-
VH-31	-	-	A	A	-	-	-	A	-	a	a	-	-	-	-	-	-	-	A	-	-
VH-32	-	-	A	A	A	a	a	-	-	a	a	-	-	-	-	-	-	-	A	-	-
VH-33	-	-	-	-	-	-	-	-	-	a	-	-	-	-	-	-	-	-	-	-	-

VH-34	-	-	-	-	-	-	-	A	-	-	-	-	-	-	-	-	-	-	-	-	-
VH-35	-	-	-	-	-	-	-	-	-	-	-	-	-	A	A	-	-	-	-	-	-
VH-36	-	-	-	-	-	-	-	A	-	-	-	-	-	-	-	-	-	-	-	-	-
VH-37	-	-	A	A	A	a	a	-	-	a	-	-	-	-	-	-	-	-	A	-	-
VH-38	D	D	A	A	-	a	-	-	-	-	-	-	-	A	A	-	-	-	-	-	-
VH-39	D	-	A	A	-	a	-	-	-	-	-	-	-	-	-	-	-	-	-	-	-
VH-40	-	-	A	-	-	-	-	-	-	a	-	-	-	-	-	-	-	-	-	-	-
VH-41	-	-	A	A	-	a	-	-	-	-	-	-	-	-	-	-	-	-	-	-	-
VH-42	-	-	-	-	-	-	-	A	-	-	-	-	-	A	-	-	-	-	-	-	-
VH-43	-	-	A	-	-	a	-	-	-	a	-	-	-	-	-	-	-	-	-	-	-
VH-44	-	-	-	-	-	-	-	-	-	-	-	-	-	-	-	-	-	D	-	-	-
VH-45	-	-	-	-	-	-	-	-	-	-	-	-	-	A	-	-	-	-	-	-	-
VH-46	-	-	-	-	-	-	-	-	-	-	-	-	-	A	-	-	-	-	-	-	-
VH-47	-	-	-	-	-	-	-	-	-	-	-	-	-	A	-	-	-	-	-	-	-
VH-48	-	-	A	A	-	-	-	-	-	-	-	-	-	-	-	-	-	-	-	-	-
VH-49	-	-	-	-	-	-	-	-	-	-	-	-	-	-	-	-	-	-	-	-	R
VH-50	-	-	-	-	-	-	-	-	-	-	-	-	R	-	-	A	-	-	-	-	-
VH-51	-	-	A	-	A	a	a	A	-	a	-	-	-	-	-	-	-	-	-	-	-
VH-52	-	-	A	-	-	a	a	-	-	-	-	-	-	-	-	-	-	-	-	-	-
VH-53	-	-	A	-	-	-	-	-	-	-	-	-	-	-	-	-	-	-	-	-	-
VH-54	-	-	-	-	-	-	-	-	-	a	-	-	R	-	-	-	-	-	-	-	-
VH-55	-	-	A	-	-	a	-	-	-	-	-	-	-	-	-	-	-	-	-	-	-
VH-56	-	-	A	-	-	-	-	-	-	-	-	-	R	-	-	A	-	-	A	-	-
VH-57	-	-	-	-	-	-	-	-	-	a	a	-	-	-	-	-	-	-	-	-	-
VH-58	-	-	-	-	-	-	-	-	-	a	-	-	-	-	-	-	-	-	-	-	-
VH-59	-	-	-	-	-	-	-	-	-	a	-	-	-	-	-	-	-	-	-	-	-
VH-60	-	-	A	A	-	-	-	-	-	a	a	-	-	-	-	-	-	-	-	-	-
VH-61	-	-	-	-	-	-	-	-	-	-	-	-	-	A	-	-	-	-	-	-	-
VH-62	-	-	-	-	-	-	-	-	-	-	-	-	-	-	-	-	-	-	A	-	-
VH-63	-	-	-	-	-	-	-	-	-	-	-	-	-	A	A	-	-	-	-	-	-
VH-64	-	-	A	A	-	a	-	-	-	-	-	-	-	-	-	-	-	-	-	-	-
VH-65	-	-	A	A	-	a	-	-	-	-	-	-	-	-	-	-	-	-	-	-	-
VH-66	-	-	-	-	-	-	-	-	-	a	-	-	-	-	-	-	-	-	-	-	-
VH-67	-	-	A	-	-	a	a	-	-	-	-	-	-	A	-	-	-	-	-	-	-
VH-68	-	-	A	A	-	-	-	-	-	a	-	-	-	-	-	-	-	-	-	-	-
VH-69	-	-	A	A	-	-	-	-	-	a	a	-	-	-	-	-	-	-	A	-	-
VH-70	-	-	-	-	-	-	-	-	-	-	-	-	-	A	A	-	-	-	-	-	-
VH-71	-	-	A	-	-	-	-	-	-	a	-	-	-	-	-	-	R	-	-	-	-
VH-72	-	-	A	A	-	a	-	-	-	a	-	-	-	-	-	-	-	-	-	-	-
VH-73	-	-	-	-	-	-	-	-	-	-	-	-	-	-	-	A	-	-	A	-	-
VH-74	-	-	-	-	-	-	-	-	-	a	-	-	-	-	-	-	-	-	-	-	-
VH-75	-	-	-	-	-	-	-	-	-	-	-	-	-	-	-	A	-	-	-	-	-
VH-76	-	-	-	-	-	-	-	A	-	-	-	-	-	-	-	-	-	-	-	-	-
VH-77	-	-	-	-	-	-	-	-	-	a	-	-	-	-	-	-	-	-	-	-	-

VH-78	-	-	A	A	-	-	-	-	-	a	a	-	-	-	-	A	-	-	-	-	-
VH-79	-	-	A	-	-	-	-	-	-	-	-	-	-	-	-	A	-	-	-	-	-
VH-80	-	-	-	-	-	-	-	-	-	-	-	A	-	-	-	-	-	-	-	-	-
VH-81	-	-	-	-	-	-	-	-	-	a	-	-	-	-	-	-	-	-	-	-	-
VH-82	-	-	-	-	-	-	-	-	-	a	a	-	-	-	-	-	-	-	-	-	-
VH-83	-	-	A	A	-	a	-	-	-	-	-	-	-	-	-	-	-	-	-	-	-
VH-84	-	-	-	-	-	-	-	-	-	-	-	-	-	-	-	A	-	-	-	-	-
VH-85	-	-	-	-	-	-	-	-	-	-	-	-	-	-	-	A	R	-	-	-	-
VH-86	-	-	-	-	-	-	-	-	-	-	-	A	-	-	-	-	-	-	-	-	-
VH-87	-	-	-	-	-	-	-	-	-	a	-	-	-	-	-	-	-	-	-	-	-
VH-88	-	-	-	-	-	-	-	-	-	-	-	-	-	A	-	-	-	-	-	-	-
VH-89	-	-	-	-	-	-	-	-	-	-	-	-	R	-	-	-	-	-	-	-	-
VH-90	-	-	A	A	-	a	-	-	-	-	-	-	R	-	-	-	-	-	-	-	-
VH-91	-	-	-	-	-	-	-	-	-	-	-	-	-	-	-	-	-	D	-	-	-
VH-92	-	-	-	-	-	-	-	-	-	-	-	-	-	A	-	-	-	-	-	-	-
VH-93	-	-	-	-	-	-	-	-	-	-	-	-	-	A	-	-	-	-	-	-	-
VH-94	-	-	-	-	-	-	-	-	-	-	-	-	-	A	-	-	-	-	-	-	-
VH-95	-	-	A	A	-	a	-	-	-	-	-	-	-	-	-	-	-	-	-	-	-
VH-96	-	-	-	-	-	-	-	-	-	-	-	-	-	-	-	A	-	-	-	-	-
VH-97	-	-	-	-	-	-	-	-	-	-	-	-	-	-	-	-	-	-	A	-	-
VH-98	D	-	-	-	-	-	-	-	-	-	-	-	-	-	-	A	-	-	-	-	-
VH-99	-	-	A	-	-	a	a	-	-	-	-	-	-	-	-	-	-	-	-	-	-
VH-100	-	-	A	-	-	-	-	-	-	-	-	-	-	-	-	-	-	-	-	-	-
VH-101	-	-	-	-	-	-	-	-	-	-	-	-	-	-	-	A	-	-	-	-	-
VH-102	-	-	-	-	-	-	-	-	-	-	-	-	-	-	-	A	-	-	-	-	-
VH-103	-	-	-	-	-	-	-	-	-	-	-	-	-	-	-	A	-	-	A	A	-
VH-104	-	-	A	-	-	-	-	-	-	a	-	-	-	-	-	-	-	-	A	A	-
VH-105	-	-	A	-	-	-	-	-	-	a	a	-	-	-	-	-	-	-	-	-	-
VH-106	-	-	A	-	-	-	-	-	-	a	a	-	-	-	-	-	-	-	-	-	-
VH-107	D	D	-	-	-	-	-	-	-	-	-	-	-	-	-	-	-	-	-	-	-
VH-108	-	-	A	A	-	-	-	-	-	a	-	-	-	A	-	-	-	-	-	-	-
VH-109	-	-	A	-	-	a	a	-	-	-	-	-	-	-	-	-	-	-	-	-	-
VH-110	-	-	-	-	-	-	-	-	-	a	-	-	-	-	-	A	-	-	-	-	-
VH-111	-	-	-	-	-	-	-	-	-	a	-	-	-	-	-	-	-	-	-	-	-
VH-112	-	-	A	-	-	-	-	-	-	a	a	-	-	-	-	-	-	-	-	-	-
VH-113	-	-	A	-	A	a	a	-	-	-	-	-	-	-	-	A	-	-	-	-	-
VH-114	-	-	A	-	-	-	-	-	-	-	-	-	-	-	-	-	-	-	-	-	-
VH-115	-	-	-	-	-	-	-	-	-	-	-	-	-	A	-	-	-	-	-	-	-
VH-116	-	-	-	-	-	-	-	-	-	-	-	-	-	-	-	-	-	D	-	-	-
VH-117	-	-	A	A	-	a	-	-	-	-	-	-	-	-	-	-	-	-	-	-	-
VH-118	-	-	A	-	-	a	-	-	-	-	-	-	-	-	-	-	-	-	-	-	-
VH-119	-	-	-	-	-	-	-	-	-	-	-	-	-	-	-	A	-	-	-	-	-
VH-120	-	-	-	-	-	-	-	-	-	-	-	-	-	-	-	-	-	-	A	-	-
VH-121	-	-	A	A	-	a	-	-	-	-	-	-	R	-	-	-	-	-	-	-	-
VH-122	-	-	A	-	A	a	a	-	-	-	-	-	-	-	-	-	-	-	-	-	-

VH-123	-	-	A	-	-	a	-	-	-	-	-	-	-	-	-	-	-	-	-	-	-
VH-124	-	-	-	-	-	a	a	-	-	-	-	-	-	A	-	-	-	-	-	-	-
VH-125	-	-	A	A	-	a	-	-	-	-	-	-	-	-	-	-	R	-	-	-	-
VH-126	-	-	-	-	-	-	-	-	-	-	-	-	-	-	-	A	-	-	-	-	-
VH-127	-	-	-	-	-	-	-	-	-	-	-	-	-	-	-	-	-	-	A	-	-
VH-128	-	-	A	-	-	-	-	-	-	a	a	-	-	-	-	-	R	-	-	-	-
VH-129	-	-	A	A	-	a	-	-	-	-	-	-	-	-	-	-	-	-	-	-	-
VH-130	-	-	A	-	-	-	-	-	-	a	a	-	-	-	-	-	-	-	A	-	-
VH-131	-	-	-	-	-	-	-	A	A	-	-	-	-	A	-	-	-	-	-	-	-
VH-132	-	-	-	-	-	-	-	-	-	-	-	-	-	-	-	A	-	-	-	-	-
VH-133	-	-	-	-	-	-	-	-	-	-	-	-	-	-	-	-	-	-	A	-	-
VH-134	-	-	-	-	-	-	-	-	-	a	a	-	-	-	-	-	-	-	-	-	-
VH-135	-	-	-	-	-	-	-	-	-	-	-	-	-	-	-	A	-	-	-	-	-
VH-136	-	-	A	-	-	-	-	-	-	a	a	-	-	-	-	-	-	-	-	-	-
VH-137	-	-	-	-	-	-	-	A	A	-	-	-	-	-	-	-	-	-	-	-	-
VH-138	-	-	-	-	-	-	-	-	-	-	-	-	-	-	-	-	-	-	A	-	-
VH-139	-	-	A	-	A	a	a	A	-	a	a	-	-	-	-	-	-	-	-	-	-
VH-140	-	-	-	-	-	-	-	A	A	-	-	-	-	-	-	-	-	-	-	-	-
VH-141	-	-	-	-	A	-	-	A	A	-	-	A	R	-	-	A	-	-	-	-	-
VH-142	-	-	-	-	-	-	-	A	A	-	-	-	-	-	-	A	-	-	-	-	-
VH-143	-	-	-	-	-	-	-	-	-	a	-	-	-	-	-	-	-	-	-	-	-
VH-144	-	-	A	A	-	a	-	-	-	-	-	-	-	-	-	-	-	-	-	-	-
VH-145	-	-	-	-	-	-	-	-	-	-	-	-	-	-	-	-	R	-	A	-	-
VH-146	-	-	-	-	-	-	-	-	-	-	-	-	-	-	-	A	-	-	A	-	-
VH-147	-	-	-	-	-	-	-	-	-	-	-	-	-	-	-	-	-	-	A	-	-
VH-148	-	-	-	-	-	-	-	-	-	-	-	-	-	-	-	-	-	-	A	-	-
VH-149	-	-	-	-	-	-	-	-	-	-	-	-	-	-	-	A	-	-	-	-	-
VH-150	-	-	-	-	-	-	-	-	-	-	-	-	-	-	-	A	-	-	A	-	-
VH-151	-	-	-	-	-	-	-	-	-	a	-	-	-	A	-	-	-	-	-	-	-
VH-152	-	-	-	-	-	-	-	-	-	-	-	-	-	-	-	A	-	-	-	-	-
VH-153	-	-	-	-	-	-	-	A	-	-	-	-	-	-	-	-	-	-	-	-	-
VH-154	-	-	-	-	-	-	-	-	-	-	-	-	-	-	-	A	-	-	-	-	-
VH-155	-	-	-	-	-	-	-	A	A	-	-	-	-	-	-	-	-	-	-	-	-
VH-156	-	-	-	-	-	-	-	-	-	a	-	-	-	-	-	-	-	-	-	-	-
VH-157	-	-	-	-	A	-	-	-	-	-	-	-	-	-	-	-	-	-	-	-	-
VH-158	-	-	A	A	-	-	-	-	-	-	-	-	-	-	-	-	-	-	-	-	-
VH-159	-	-	-	-	-	-	-	-	-	-	-	-	-	A	-	-	-	-	-	-	-
VH-160	-	-	A	A	-	a	-	-	-	-	-	-	-	-	-	-	-	-	-	-	-
VH-161	-	-	A	-	-	-	-	-	-	-	-	-	-	-	-	-	-	-	A	-	-
VH-162	-	-	-	-	-	-	-	-	-	-	-	-	-	-	-	-	-	-	A	-	-
VH-163	-	-	-	-	-	-	-	-	-	-	-	-	-	-	-	A	-	-	-	-	-
VH-164	-	-	A	-	-	a	a	-	-	-	-	-	-	-	-	-	-	-	-	-	-
VH-165	-	-	A	-	-	a	-	-	-	-	-	-	-	-	-	-	-	-	-	-	-
VH-166	-	-	A	A	-	a	-	-	-	a	-	-	-	-	-	-	-	-	-	-	-
VH-167	-	-	A	-	-	-	-	-	-	a	a	-	-	-	-	-	-	-	-	-	-

VH-168	-	-	-	-	-	-	-	-	-	-	-	-	-	A	-	-	-	-	-	-	-
VH-169	-	-	-	-	-	-	-	-	-	a	-	-	-	-	-	-	-	-	A	-	-
VH-170	-	-	-	-	-	-	-	-	-	-	-	-	-	-	-	-	-	-	-	-	-
VH-171	D	-	-	-	-	-	-	-	-	-	-	-	-	-	-	A	-	-	-	-	-
VH-172	-	-	-	-	-	-	-	A	-	a	-	-	-	-	-	-	-	-	-	-	-
VH-173	-	-	-	-	-	-	-	-	-	-	-	-	R	-	-	A	-	-	-	-	-
VH-174	-	-	-	-	-	-	-	-	-	a	-	-	-	-	-	A	-	-	-	-	-
VH-175	-	-	-	-	-	-	-	-	-	a	-	-	-	-	-	A	-	-	-	-	-
VH-176	-	-	A	A	-	-	-	-	-	a	a	-	-	-	-	-	-	-	-	-	-
VH-177	-	-	-	-	-	-	-	-	-	-	-	-	-	-	-	-	-	-	A	-	-
VH-178	D	D	A	-	-	a	a	-	-	-	-	-	-	-	-	-	-	-	-	-	-
VH-179	-	-	-	-	-	-	-	-	-	a	-	-	-	-	-	-	-	-	-	-	-
VH-180	-	-	-	-	-	-	-	-	-	-	-	-	-	-	-	A	-	-	-	-	-
VH-181	-	-	-	-	-	-	-	A	A	-	-	-	-	-	-	-	-	-	-	-	-
VH-182	-	-	-	-	-	-	-	-	-	a	-	-	-	-	-	-	-	-	-	-	-
VH-183	-	-	A	A	-	a	-	-	-	-	-	-	-	A	-	-	-	-	-	-	-
VH-184	-	-	A	A	-	-	-	-	-	-	-	-	-	-	-	-	-	-	-	-	-
VH-185	-	-	A	A	A	a	a	A	-	a	a	-	-	-	-	-	-	-	A	-	-
VH-186	-	-	-	-	-	-	-	-	-	-	-	-	-	-	-	A	-	-	-	-	-
VH-187	-	-	-	-	-	-	-	-	-	-	-	-	-	A	A	-	-	-	-	-	-
VH-188	-	-	A	-	-	-	-	-	-	-	-	-	-	-	-	-	-	-	-	-	-
VH-189	-	-	A	A	-	-	-	-	-	a	a	-	-	-	-	-	-	-	-	-	-
VH-190	-	-	-	-	-	-	-	-	-	a	-	-	-	-	-	-	-	-	-	-	-
VH-191	-	-	A	-	-	-	-	-	-	-	-	-	-	-	-	-	-	-	-	-	-
VH-192	-	-	A	-	-	-	-	-	-	-	-	-	-	A	-	-	-	-	-	-	-
VH-193	-	-	-	-	-	a	-	-	-	-	-	-	-	-	-	-	-	-	-	-	-
VH-194	-	-	-	-	-	a	-	-	-	a	-	-	-	-	-	-	-	-	A	-	-
VH-195	-	-	-	-	-	-	-	-	-	-	-	-	-	A	-	-	-	-	-	-	-
VH-196	-	-	A	-	-	-	-	-	-	-	-	-	-	-	-	-	-	-	-	-	-
VH-197	-	-	A	-	-	-	-	-	-	a	-	-	-	-	-	-	-	-	-	-	-
VH-198	-	-	A	A	-	-	-	-	-	a	-	-	-	A	-	-	-	-	-	-	-
VH-199	D	-	-	-	-	-	-	-	-	-	-	-	-	A	-	-	-	-	-	-	-
VH-200	-	-	-	-	A	-	-	-	-	-	-	-	-	-	-	-	-	-	-	-	-
VH-201	-	-	-	-	-	-	-	A	A	-	-	-	-	-	-	-	-	-	-	-	R
VH-202	-	-	-	-	-	-	-	-	-	-	-	-	-	-	-	A	-	-	-	-	-
VH-203	-	-	-	-	-	-	-	-	-	-	-	-	R	-	-	-	-	-	-	-	-
VH-204	-	-	A	-	A	a	-	-	-	-	-	-	-	-	-	-	-	-	-	-	-
VH-205	-	-	A	-	A	a	a	A	A	a	-	-	-	-	-	-	-	-	-	-	-
VH-206	-	-	-	-	A	a	-	-	-	-	-	-	-	-	-	-	-	-	-	-	-
VH-207	-	-	-	-	-	-	-	A	A	-	-	-	-	-	-	-	-	-	-	-	-
VH-208	-	-	-	-	-	-	-	-	-	-	-	-	-	A	-	-	-	-	-	-	-
VH-209	-	-	A	A	-	a	-	A	-	-	-	-	-	A	-	-	-	-	-	-	-
VH-210	-	-	A	-	-	-	-	-	-	a	a	-	-	A	-	-	-	-	-	-	-
VH-211	-	-	-	-	-	-	-	-	-	a	a	-	-	A	-	-	-	-	-	-	-
VH-212	-	-	A	-	-	-	-	-	-	-	-	-	-	-	-	-	-	-	-	-	-

VH-213	-	-	-	-	-	-	-	-	-	-	-	-	-	-	-	A	-	-	-	-	-
VH-214	-	-	-	-	-	-	-	-	-	-	-	-	-	-	-	A	-	-	-	-	-
VH-215	-	-	-	-	-	-	-	-	-	-	-	-	-	A	A	A	-	-	-	-	-
VH-216	-	-	-	-	-	-	-	-	-	-	-	-	-	A	A	A	-	-	A	-	-
VH-217	-	-	A	A	-	-	-	-	-	-	-	-	-	-	-	-	-	-	-	-	-
VH-218	-	-	A	A	-	a	-	-	-	-	-	-	-	-	-	A	-	-	A	A	-
VH-219	-	-	-	-	-	-	-	-	-	-	-	-	-	-	-	-	-	-	A	-	-
VH-220	-	-	A	-	A	-	-	-	-	-	-	-	-	-	-	-	-	-	-	-	-
VH-221	-	-	-	-	-	-	-	-	-	a	-	-	-	-	-	A	-	-	-	-	-
VH-222	-	-	-	-	-	-	-	-	-	-	-	-	-	-	-	A	-	-	A	-	-
VH-223	-	-	-	-	-	-	-	-	-	-	-	-	-	A	-	-	-	-	-	-	-
VH-224	-	-	A	A	-	a	-	-	-	-	-	-	-	-	-	-	-	-	-	-	-
VH-225	-	-	A	A	-	-	-	A	-	a	a	-	-	-	-	-	-	-	-	-	-
VH-226	-	-	-	-	-	-	-	-	-	-	-	-	-	-	-	A	-	-	-	-	-
VH-227	-	-	A	-	-	a	-	-	-	-	-	-	-	-	-	-	-	-	-	-	-
VH-228	-	-	-	-	-	-	-	-	-	-	-	-	-	A	-	-	-	-	A	-	-
VH-229	-	-	A	-	A	-	-	-	-	-	-	-	-	-	-	-	-	-	-	-	-
VH-230	-	-	A	A	-	-	-	-	-	a	-	-	-	-	-	-	-	-	-	-	-
VH-231	-	-	A	A	-	-	-	-	-	a	-	-	-	-	-	-	-	-	-	-	-
VH-232	-	-	-	-	-	-	-	A	-	-	-	-	-	-	-	-	-	-	-	-	-
VH-233	-	-	A	A	-	a	-	-	-	-	-	-	-	-	-	-	-	-	-	-	-
VH-234	-	-	-	-	-	-	-	A	-	-	-	-	-	-	-	A	-	-	-	-	-
VH-235	-	-	A	A	A	-	-	A	A	-	-	-	-	-	-	-	-	-	-	-	-
VH-236	-	-	A	-	-	a	-	-	-	-	-	-	-	-	-	A	-	-	A	-	-
VH-237	-	-	-	-	-	-	-	-	-	-	-	-	-	-	-	A	-	-	-	-	-
VH-238	-	-	-	-	-	-	-	-	-	-	-	-	-	-	-	A	-	-	-	-	-
VH-239	-	-	-	-	-	-	-	-	-	-	-	-	-	A	-	-	-	-	-	-	-
VH-240	-	-	A	-	-	-	-	A	A	a	-	-	-	-	-	-	-	-	-	-	-
VH-241	-	-	-	-	-	-	-	A	-	-	-	-	-	-	-	-	-	-	-	-	-
VH-242	-	-	-	-	-	-	-	-	-	-	-	-	-	-	-	-	-	-	A	-	-
VH-243	-	-	-	-	-	-	-	-	-	a	-	-	-	-	-	-	-	-	-	-	-
VH-244	D	-	-	-	-	-	-	-	-	-	-	-	-	-	-	-	-	-	-	-	-
VH-245	-	-	-	-	-	-	-	-	-	-	-	-	-	-	-	A	-	-	-	-	-
VH-246	-	-	-	-	-	-	-	-	-	-	-	-	-	-	-	A	-	-	-	-	-
VH-247	-	-	A	-	-	a	-	-	-	a	-	-	-	-	-	-	-	-	-	-	-
VH-248	-	-	-	-	-	-	-	-	-	-	-	-	-	-	-	A	R	-	-	-	-
VH-249	-	-	-	-	-	-	-	-	-	-	-	-	-	A	-	-	-	-	-	-	-
VH-250	-	-	A	A	-	-	-	-	-	a	a	-	-	-	-	-	-	-	-	-	-
VH-251	-	-	A	-	-	a	-	-	-	a	-	-	-	-	-	-	-	-	-	-	R
VH-252	-	-	-	-	-	-	-	-	-	a	-	-	-	-	-	-	-	-	-	-	-
VH-253	-	-	-	-	-	-	-	-	-	-	-	-	R	-	-	-	-	-	-	-	-
VH-254	-	-	-	-	-	-	-	-	-	-	-	-	-	-	-	-	R	-	-	-	-
VH-255	-	-	-	-	-	-	-	A	-	-	-	-	-	-	-	-	-	-	-	-	-
VH-256	-	-	-	-	-	-	-	-	-	-	-	-	-	-	-	A	-	-	-	-	-
VH-257	-	-	A	-	-	-	-	-	-	-	-	-	-	-	-	A	-	-	A	-	-

VH-258	-	-	-	-	-	-	-	-	-	-	-	-	-	A	-	A	-	-	A	A	-
VH-259	-	-	A	A	-	a	-	-	-	-	-	-	-	A	A	-	-	-	-	-	-
VH-260	-	-	A	A	-	a	-	-	-	-	-	-	-	-	-	-	-	-	A	-	-
VH-261	-	-	A	A	-	-	-	-	-	a	a	-	-	-	-	-	-	-	-	-	-
VH-262	-	-	-	-	-	-	-	-	-	-	-	-	-	-	-	A	-	-	-	-	-
VH-263	-	-	A	A	-	-	-	-	-	-	-	-	-	-	-	-	-	-	-	-	-
VH-264	-	-	-	-	-	a	a	-	-	-	-	-	-	-	-	-	-	-	-	-	-
VH-265	-	-	A	A	-	-	-	A	A	a	a	-	-	-	-	-	-	-	-	-	R
VH-266	-	-	A	A	-	-	-	-	-	-	-	-	-	-	-	-	-	-	-	-	-
VH-267	-	-	A	A	-	-	-	-	-	a	a	-	-	-	-	-	-	-	-	-	-
VH-268	-	-	-	-	-	-	-	-	-	-	-	-	-	-	-	A	-	-	-	-	-
VH-269	-	-	A	-	-	-	-	-	-	a	-	-	-	A	A	-	-	-	-	-	-
VH-270	-	-	A	-	-	-	-	-	-	a	-	-	-	A	A	-	-	-	-	-	-
VH-271	-	-	-	-	-	-	-	-	-	a	-	-	-	-	-	-	-	-	-	-	-
VH-272	-	-	-	-	-	-	-	-	-	a	-	-	-	-	-	-	-	-	-	-	-
VH-273	-	-	-	-	-	-	-	-	-	a	-	A	-	-	-	-	-	-	-	-	-
VH-274	-	-	-	-	-	a	-	-	-	-	-	-	-	-	-	-	-	-	-	-	-
VH-275	-	-	A	A	-	-	-	A	-	a	a	-	-	-	-	-	-	-	-	-	-

4. Table S2: Novartis diverse library HTRF screen result dataset

Using the compound library annotated in the reference 55 [Schuffenhauer A, Schneider N, Hintermann S, et al (2020) Evolution of Novartis' Small Molecule Screening Deck Design. J Med Chem 63:14425–14447. https://doi.org/10.1021/acs.jmedchem.0c01332], Novartis diverse library HTRF screen tested 1,101,793 compounds at 40 μM. Here is the compound number distribution table across each % inhibition bin to represent the dataset.

x: % inhibition at 40 μM (bin)	Compound Number
x < -40.00 (hit)	2715
-40.00 ≤ x < -30.00	3244
-30.00 ≤ x < -20.00	12209
-20.00 ≤ x < -10.00	63595
-10.00 ≤ x < 0.00	409873
0.00 ≤ x < 10.00	486815
10.00 ≤ x < 20.00	100861
20.00 ≤ x < 30.00	14396
30.00 ≤ x < 40.00	3257
x ≥ 40	4828

5. Table S3: ChEMBL compound set screen HTRF result

Example dataset of 751 ChEMBL compounds (ChEMBL version 20 dataset: https://ftp.ebi.ac.uk/pub/databases/chembl/ChEMBLdb/releases/chembl_20/) were tested at 40 μM with the IDs indicated in the table. WDR5 HTRF activity (% inhibition) was shown for both replicates in the screen and inhibition > -40.00% was used to call hits (CHEMBL1081548 and CHEMBL1570577). CHEMBL1081548 was selected to further confirm its activity by dose response curve.

chembl_id	replicate 1	replicate 2	chembl_id	replicate 1	replicate 2	chembl_id	replicate 1	replicate 2
WM-662 (reference)	-65.21	-64.83	CHEMBL486179	-3.09	-4.80	CHEMBL1608977	0.60	4.50
CHEMBL1081548	-45.23	-40.12	CHEMBL1413761	-3.08	-5.60	CHEMBL1881197	0.62	0.42
CHEMBL1570577	-40.58	-12.33	CHEMBL1487283	-3.06	3.23	CHEMBL1311109	0.63	5.17
CHEMBL1591962	-26.18	2.56	CHEMBL1727392	-3.02	2.62	CHEMBL1548446	0.63	1.10
CHEMBL1562616	-17.60	-24.51	CHEMBL1343411	-3.00	-0.21	CHEMBL1365669	0.66	-0.28
CHEMBL1434896	-9.68	-21.01	CHEMBL1623290	-2.96	-5.02	CHEMBL1381184	0.69	0.25
CHEMBL3276818	-19.77	6.66	CHEMBL1580813	-2.93	-1.02	CHEMBL1342629	0.71	-3.61
CHEMBL1472842	-19.45	-14.06	CHEMBL171195	-2.92	-0.18	CHEMBL1551413	0.72	1.52
CHEMBL1553369	-18.65	-13.73	CHEMBL587174	-2.89	3.13	CHEMBL264472	0.73	-0.81
CHEMBL244609	-18.31	-8.20	CHEMBL1466273	-2.89	3.36	CHEMBL563451	0.73	1.51
CHEMBL1537560	-17.11	-9.43	CHEMBL1479791	-2.89	2.55	CHEMBL1413518	0.73	2.76
CHEMBL1331558	-17.06	-9.56	CHEMBL2095013	-2.88	-0.65	CHEMBL1327970	0.76	6.69
CHEMBL1531188	-16.45	-17.68	CHEMBL1528099	-2.87	-4.73	CHEMBL597874	0.77	-0.26
CHEMBL1470017	-15.79	-9.37	CHEMBL1502775	-2.86	-1.43	CHEMBL1256682	0.80	2.17
CHEMBL1547762	-15.23	-9.60	CHEMBL1335789	-2.84	5.19	CHEMBL1499907	0.81	-1.73
CHEMBL1398888	-15.12	-17.20	CHEMBL1866221	-2.81	-0.65	CHEMBL1510863	0.82	-3.50
CHEMBL1621440	-14.75	-10.73	CHEMBL1506065	-2.78	-0.48	CHEMBL1332367	0.84	0.16
CHEMBL381433	-14.24	-16.29	CHEMBL1552347	-2.78	2.68	CHEMBL1441374	0.84	-0.33
CHEMBL1624903	-13.69	-11.60	CHEMBL1316477	-2.72	-6.76	CHEMBL1486684	0.88	-12.05
CHEMBL1474827	-13.68	-11.15	CHEMBL1350621	-2.72	-1.99	CHEMBL10623	0.92	-1.81
CHEMBL1703705	-12.88	-9.03	CHEMBL1398395	-2.68	4.88	CHEMBL1453776	0.93	5.90
CHEMBL1622178	-12.56	-15.96	CHEMBL1704458	-2.65	-0.59	CHEMBL1305646	0.93	2.55
CHEMBL1330082	-12.46	-14.98	CHEMBL1869801	-2.65	2.20	CHEMBL1588367	0.93	10.30
CHEMBL1427383	-12.16	-10.02	CHEMBL1561644	-2.58	-0.17	CHEMBL1327076	0.94	6.70
CHEMBL1315282	-12.12	-5.35	CHEMBL1502519	-2.58	-5.36	CHEMBL466565	0.94	4.30
CHEMBL1304324	-12.08	-9.22	CHEMBL1312979	-2.55	8.27	CHEMBL1559524	0.94	-1.85
CHEMBL1482649	-11.98	-14.91	CHEMBL1393413	-2.55	-3.69	CHEMBL1440435	0.95	-1.40
CHEMBL1557732	-11.92	-15.64	CHEMBL1481931	-2.54	-0.64	CHEMBL1605452	0.98	2.82
CHEMBL1300046	-11.68	-10.54	CHEMBL1729202	-2.53	1.93	CHEMBL1431620	0.98	6.10
CHEMBL1482269	-11.44	-6.29	CHEMBL1542074	-2.53	2.67	CHEMBL1438245	0.99	3.63
CHEMBL1432954	-11.17	-11.13	CHEMBL1391162	-2.48	-3.04	CHEMBL1481572	1.02	3.73
CHEMBL1304672	-11.11	3.89	CHEMBL1609803	-2.47	-3.05	CHEMBL1480947	1.03	3.17
CHEMBL1444774	-10.90	-11.02	CHEMBL1735676	-2.47	-4.31	CHEMBL1864950	1.05	1.08
CHEMBL1574618	-10.60	-5.55	CHEMBL3109592	-2.46	5.01	CHEMBL1366843	1.05	-5.01
CHEMBL74432	-10.56	-7.71	CHEMBL1550577	-2.43	4.13	CHEMBL2143997	1.05	0.26
CHEMBL1524855	-10.56	-6.51	CHEMBL3182065	-2.41	-6.85	CHEMBL1476340	1.05	-0.46
CHEMBL3208767	-10.32	-10.78	CHEMBL1885570	-2.40	0.81	CHEMBL1354471	1.06	-0.75
CHEMBL1897197	-10.26	-3.18	CHEMBL1619140	-2.40	-1.65	CHEMBL2003406	1.07	-0.25
CHEMBL1444414	-10.02	-7.57	CHEMBL43002	-2.40	1.14	CHEMBL1307516	1.08	2.94

CHEMBL75841	-10.00	-8.96	CHEMBL2107472	-2.39	0.15	CHEMBL1300075	1.08	-9.83
CHEMBL2041334	-9.97	-11.01	CHEMBL1703859	-2.39	1.20	CHEMBL1734851	1.11	2.23
CHEMBL1699877	-9.95	-3.34	CHEMBL527585	-2.34	-2.53	CHEMBL1305377	1.11	1.37
CHEMBL1377765	-9.92	-10.18	CHEMBL1457921	-2.33	1.95	CHEMBL1534355	1.12	-0.28
CHEMBL1388288	-9.90	-16.95	CHEMBL1603583	-2.31	-2.42	CHEMBL1524186	1.12	1.06
CHEMBL1396334	-9.85	-6.75	CHEMBL1605337	-2.25	0.15	CHEMBL340211	1.15	9.80
CHEMBL1323554	-9.85	-5.08	CHEMBL1560437	-2.24	-2.50	CHEMBL1882377	1.15	5.51
CHEMBL1529845	-9.78	-7.10	CHEMBL1396820	-2.22	-2.31	CHEMBL1404076	1.16	3.31
CHEMBL1490937	-9.71	-5.56	CHEMBL1532396	-2.20	3.51	CHEMBL1521731	1.20	1.13
CHEMBL1407617	-9.60	-1.59	CHEMBL1401418	-2.19	-4.47	CHEMBL1316881	1.26	-2.95
CHEMBL1379624	-9.53	-6.37	CHEMBL2286384	-2.18	-1.62	CHEMBL1419944	1.27	0.97
CHEMBL1564595	-9.50	1.25	CHEMBL1606478	-2.16	-1.05	CHEMBL2358757	1.27	6.18
CHEMBL1364486	-9.49	-2.42	CHEMBL1326663	-2.15	-0.76	CHEMBL1622927	1.28	3.57
CHEMBL1613630	-9.44	-17.22	CHEMBL1356692	-2.14	-4.83	CHEMBL1444686	1.29	-4.12
CHEMBL2360057	-9.37	-10.79	CHEMBL1352107	-2.14	-1.04	CHEMBL1368227	1.34	0.38
CHEMBL1706320	-9.34	4.45	CHEMBL1320326	-2.13	-0.71	CHEMBL1345751	1.34	-2.28
CHEMBL1742267	-9.29	2.24	CHEMBL1371914	-2.10	4.03	CHEMBL1346469	1.35	-5.75
CHEMBL1915725	-9.10	-5.44	CHEMBL1355169	-2.09	-6.20	CHEMBL235741	1.44	4.96
CHEMBL1624629	-8.98	-6.69	CHEMBL1578520	-2.07	0.15	CHEMBL1302058	1.45	1.38
CHEMBL1616575	-8.94	-10.69	CHEMBL1445228	-2.06	3.72	CHEMBL1345088	1.46	2.23
CHEMBL1487933	-8.94	-10.40	CHEMBL1460333	-2.04	1.75	CHEMBL1441853	1.47	3.14
CHEMBL1471623	-8.94	-17.87	CHEMBL1405793	-2.04	-1.09	CHEMBL1561613	1.48	2.35
CHEMBL1567716	-8.78	-0.88	CHEMBL1902583	-2.02	0.95	CHEMBL3210958	1.49	3.10
CHEMBL1526449	-8.64	-9.49	CHEMBL1703671	-2.01	-1.22	CHEMBL2361914	1.50	-3.40
CHEMBL1581005	-8.62	-6.07	CHEMBL1554534	-2.01	0.13	CHEMBL1872103	1.50	4.68
CHEMBL2360435	-8.60	-9.27	CHEMBL1625276	-2.00	-0.32	CHEMBL1409465	1.50	6.25
CHEMBL1345475	-8.59	-0.33	CHEMBL1394788	-1.98	4.01	CHEMBL1722425	1.52	2.12
CHEMBL1452045	-8.49	-0.72	CHEMBL2237491	-1.94	-0.27	CHEMBL1488209	1.54	5.37
CHEMBL1452327	-8.46	-12.44	CHEMBL1484677	-1.93	4.89	CHEMBL170047	1.60	3.78
CHEMBL1327876	-8.34	-1.57	CHEMBL1473402	-1.93	1.92	CHEMBL1735579	1.61	0.52
CHEMBL1332771	-8.32	5.68	CHEMBL1391644	-1.91	-11.30	CHEMBL1473070	1.62	2.88
CHEMBL1423200	-8.31	-8.23	CHEMBL1360911	-1.87	-5.73	CHEMBL1616556	1.62	2.12
CHEMBL1367852	-8.20	-10.90	CHEMBL1505504	-1.84	-2.61	CHEMBL1570709	1.63	-4.17
CHEMBL1564097	-8.16	1.48	CHEMBL422289	-1.81	8.11	CHEMBL470442	1.71	7.44
CHEMBL3187738	-8.07	-6.37	CHEMBL1445383	-1.80	3.80	CHEMBL1703865	1.74	-2.00
CHEMBL1308240	-8.02	-2.69	CHEMBL1480975	-1.80	5.97	CHEMBL46525	1.75	-2.58
CHEMBL1496871	-8.01	-5.15	CHEMBL589435	-1.77	2.94	CHEMBL1487155	1.80	4.05
CHEMBL577807	-7.97	-3.55	CHEMBL1436281	-1.71	2.48	CHEMBL1492800	1.82	2.27
CHEMBL1864356	-7.94	-0.73	CHEMBL1357807	-1.71	-0.12	CHEMBL1316347	1.87	-4.76
CHEMBL1728301	-7.94	-5.53	CHEMBL1622426	-1.70	-2.14	CHEMBL1463569	1.87	3.37
CHEMBL1735562	-7.84	-6.76	CHEMBL1448172	-1.70	0.15	CHEMBL1621061	1.90	0.10
CHEMBL1317240	-7.77	2.02	CHEMBL1577895	-1.66	-0.42	CHEMBL1333142	1.91	2.76
CHEMBL1741134	-7.71	-5.28	CHEMBL1863987	-1.65	-3.02	CHEMBL1907583	1.92	1.53
CHEMBL1560218	-7.59	-6.90	CHEMBL1473742	-1.64	3.41	CHEMBL585640	1.92	-1.28
CHEMBL609899	-7.52	-1.88	CHEMBL1455579	-1.61	3.16	CHEMBL1916377	1.93	1.82
CHEMBL1301716	-7.52	-9.67	CHEMBL1400474	-1.61	8.44	CHEMBL1621180	1.96	1.48
CHEMBL1541219	-7.44	-1.51	CHEMBL1428855	-1.61	-4.07	CHEMBL562318	1.98	1.27
CHEMBL1429923	-7.41	-12.62	CHEMBL1495368	-1.60	1.90	CHEMBL1394175	2.02	2.57
CHEMBL418907	-7.41	-6.48	CHEMBL1200761	-1.60	-0.17	CHEMBL1196311	2.04	-1.20

CHEMBL1410729	-7.40	-2.79	CHEMBL1419720	-1.59	-0.39	CHEMBL1414588	2.07	5.98
CHEMBL1431233	-7.34	-8.02	CHEMBL1317107	-1.59	-2.01	CHEMBL1895719	2.10	2.08
CHEMBL1519642	-7.27	-0.64	CHEMBL1554174	-1.55	0.75	CHEMBL1604381	2.14	3.71
CHEMBL2141755	-7.25	-5.91	CHEMBL1600003	-1.53	-2.09	CHEMBL1564115	2.15	0.26
CHEMBL1383591	-7.20	-5.86	CHEMBL1897427	-1.53	3.67	CHEMBL1549507	2.19	7.38
CHEMBL1709494	-7.10	-7.52	CHEMBL3260512	-1.50	-2.70	CHEMBL1290589	2.20	11.36
CHEMBL227925	-7.09	-3.41	CHEMBL1403015	-1.49	0.27	CHEMBL1710460	2.25	8.09
CHEMBL1339632	-6.90	-4.20	CHEMBL3260515	-1.49	-4.38	CHEMBL2289333	2.26	1.01
CHEMBL1382213	-6.89	-7.09	CHEMBL1385719	-1.47	1.58	CHEMBL1893868	2.26	-1.85
CHEMBL1321474	-6.88	-4.53	CHEMBL1493280	-1.40	8.23	CHEMBL1384870	2.32	2.15
CHEMBL1348501	-6.86	0.56	CHEMBL1896952	-1.37	5.50	CHEMBL1491038	2.39	-2.71
CHEMBL1400492	-6.79	1.24	CHEMBL1592443	-1.36	-1.75	CHEMBL1620739	2.39	-1.37
CHEMBL1605120	-6.68	2.97	CHEMBL1313485	-1.33	-1.83	CHEMBL1565765	2.42	6.04
CHEMBL492234	-6.65	-5.64	CHEMBL1493319	-1.29	6.24	CHEMBL1410868	2.47	-2.71
CHEMBL1612646	-6.64	-7.13	CHEMBL1608317	-1.27	-0.48	CHEMBL1492829	2.49	-0.25
CHEMBL1339771	-6.61	-0.86	CHEMBL1450236	-1.27	-4.34	CHEMBL1387239	2.50	4.77
CHEMBL1376249	-6.61	-7.85	CHEMBL1863787	-1.26	3.03	CHEMBL1423803	2.51	0.18
CHEMBL1594611	-6.47	-0.10	CHEMBL1880071	-1.25	-11.47	CHEMBL1365765	2.52	-0.10
CHEMBL1448966	-6.44	-1.12	CHEMBL1566084	-1.23	3.85	CHEMBL1872474	2.53	6.45
CHEMBL1441303	-6.41	-1.66	CHEMBL1521929	-1.20	-4.00	CHEMBL1456478	2.56	7.87
CHEMBL1474026	-6.38	-3.89	CHEMBL1328134	-1.19	6.10	CHEMBL2359140	2.57	3.01
CHEMBL1532257	-6.37	-7.83	CHEMBL1338903	-1.16	-0.61	CHEMBL1896333	2.58	4.67
CHEMBL1865304	-6.30	-6.29	CHEMBL1940414	-1.15	-0.68	CHEMBL1371414	2.63	3.58
CHEMBL1477278	-6.25	-4.02	CHEMBL1544573	-1.15	2.92	CHEMBL1995148	2.63	1.15
CHEMBL1429787	-6.25	-0.03	CHEMBL1896305	-1.12	-0.72	CHEMBL1341037	2.65	9.34
CHEMBL1492219	-6.23	-2.28	CHEMBL1493663	-1.11	1.17	CHEMBL1325280	2.66	2.65
CHEMBL2107589	-6.22	-3.74	CHEMBL1450078	-1.06	-2.51	CHEMBL1400780	2.68	-3.37
CHEMBL1356937	-6.14	0.23	CHEMBL1512937	-1.05	1.40	CHEMBL1486255	2.73	-2.29
CHEMBL1617756	-6.11	-3.63	CHEMBL1711473	-1.00	9.20	CHEMBL1443010	2.74	-4.96
CHEMBL1437572	-5.96	-3.27	CHEMBL1603965	-0.98	1.43	CHEMBL1882491	2.74	0.29
CHEMBL1508317	-5.87	-2.09	CHEMBL1474141	-0.96	0.26	CHEMBL1645045	2.74	14.04
CHEMBL1482089	-5.86	-0.62	CHEMBL1314027	-0.95	-2.29	CHEMBL1343365	2.79	1.12
CHEMBL44767	-5.84	-4.74	CHEMBL1893696	-0.94	3.35	CHEMBL1337117	2.82	1.36
CHEMBL1408909	-5.78	2.88	CHEMBL1495954	-0.92	6.06	CHEMBL1500095	2.83	2.78
CHEMBL1528154	-5.77	-8.72	CHEMBL1892033	-0.92	8.70	CHEMBL89253	2.85	0.69
CHEMBL1353856	-5.73	-0.98	CHEMBL1864870	-0.92	-0.48	CHEMBL1494366	2.87	8.44
CHEMBL288034	-5.71	4.15	CHEMBL3260506	-0.91	2.83	CHEMBL1534916	2.89	-0.77
CHEMBL1418273	-5.69	-1.71	CHEMBL1426161	-0.91	6.10	CHEMBL1299863	2.89	0.30
CHEMBL1438353	-5.63	2.95	CHEMBL1619405	-0.88	-4.42	CHEMBL1468393	2.93	0.25
CHEMBL2141397	-5.59	-6.55	CHEMBL1318382	-0.86	5.17	CHEMBL2093356	2.93	4.73
CHEMBL1360667	-5.55	-1.04	CHEMBL1312229	-0.85	1.74	CHEMBL578912	2.93	-0.76
CHEMBL1612571	-5.55	-4.21	CHEMBL1522092	-0.83	5.90	CHEMBL1425424	2.96	3.61
CHEMBL1406932	-5.52	-9.77	CHEMBL1375726	-0.82	5.29	CHEMBL1534743	2.97	-4.34
CHEMBL3190620	-5.50	2.89	CHEMBL1443655	-0.82	0.90	CHEMBL1351376	3.00	-0.76
CHEMBL2132756	-5.46	0.04	CHEMBL1412238	-0.81	2.31	CHEMBL564460	3.02	-1.78
CHEMBL269154	-5.42	-2.48	CHEMBL1358745	-0.77	0.48	CHEMBL1431862	3.02	1.17
CHEMBL1310826	-5.40	-5.74	CHEMBL337745	-0.77	-2.57	CHEMBL2135474	3.03	-1.33
CHEMBL1357131	-5.37	-4.15	CHEMBL1442979	-0.76	4.12	CHEMBL1351000	3.07	3.89
CHEMBL1722621	-5.36	4.45	CHEMBL1201216	-0.76	2.70	CHEMBL1549742	3.10	5.10

CHEMBL1540597	-5.31	-9.47	CHEMBL3183750	-0.71	5.35	CHEMBL1373301	3.11	-0.19
CHEMBL1422550	-5.26	-3.91	CHEMBL1742452	-0.70	3.60	CHEMBL1550551	3.22	-0.37
CHEMBL1517016	-5.25	-2.27	CHEMBL137119	-0.69	-2.98	CHEMBL1302566	3.24	4.24
CHEMBL1594855	-5.22	-5.46	CHEMBL1546262	-0.69	3.12	CHEMBL2152526	3.26	2.06
CHEMBL1306088	-5.20	3.23	CHEMBL1875112	-0.69	-16.99	CHEMBL1442350	3.30	2.36
CHEMBL576797	-5.18	-3.62	CHEMBL1486554	-0.66	-5.25	CHEMBL1356515	3.33	-2.59
CHEMBL1617494	-5.17	-4.99	CHEMBL1337747	-0.64	4.16	CHEMBL1366410	3.35	-6.23
CHEMBL234994	-5.17	-3.76	CHEMBL1473380	-0.63	3.68	CHEMBL1620454	3.35	2.37
CHEMBL1346781	-5.12	-6.67	CHEMBL1599246	-0.63	0.19	CHEMBL1720136	3.36	-0.30
CHEMBL3196081	-5.12	-2.21	CHEMBL1444799	-0.63	2.55	CHEMBL1709868	3.38	4.98
CHEMBL1302466	-5.10	1.85	CHEMBL1494205	-0.61	2.28	CHEMBL1594834	3.41	6.48
CHEMBL1733955	-5.08	-4.83	CHEMBL1426601	-0.61	-2.98	CHEMBL1455112	3.44	4.49
CHEMBL1375169	-5.08	-0.24	CHEMBL1368344	-0.60	2.14	CHEMBL1645043	3.46	0.44
CHEMBL1353511	-5.05	-2.38	CHEMBL1390532	-0.59	-3.80	CHEMBL1391360	3.53	4.64
CHEMBL2234893	-5.03	-5.50	CHEMBL1618362	-0.59	-2.25	CHEMBL1599783	3.54	1.58
CHEMBL1479446	-5.03	-2.22	CHEMBL1301122	-0.58	5.36	CHEMBL1521561	3.60	-1.41
CHEMBL1880144	-5.01	2.56	CHEMBL1721460	-0.57	-3.91	CHEMBL590215	3.62	7.99
CHEMBL2093339	-4.96	1.71	CHEMBL1334923	-0.56	3.50	CHEMBL1402194	3.70	7.43
CHEMBL15112	-4.96	0.49	CHEMBL83727	-0.55	-4.03	CHEMBL1878904	3.79	-1.14
CHEMBL1372771	-4.92	-2.81	CHEMBL475207	-0.55	5.68	CHEMBL2110645	3.80	2.52
CHEMBL1316696	-4.88	-6.92	CHEMBL1551130	-0.55	4.00	CHEMBL1607475	3.85	5.28
CHEMBL1889300	-4.86	-2.81	CHEMBL1466283	-0.54	2.72	CHEMBL1901451	3.85	3.36
CHEMBL1897385	-4.79	5.57	CHEMBL1588943	-0.53	5.18	CHEMBL1540638	3.87	5.81
CHEMBL1351395	-4.78	-3.34	CHEMBL1880950	-0.53	-5.84	CHEMBL3104902	3.99	-4.17
CHEMBL1719512	-4.73	-7.45	CHEMBL1360956	-0.53	1.37	CHEMBL1572517	4.02	-0.23
CHEMBL1406386	-4.72	-1.78	CHEMBL1535650	-0.50	1.05	CHEMBL1609770	4.10	5.75
CHEMBL1572962	-4.72	0.14	CHEMBL3209890	-0.50	-2.10	CHEMBL1439678	4.15	8.94
CHEMBL1699200	-4.67	0.64	CHEMBL1318193	-0.50	-2.75	CHEMBL1359795	4.21	0.64
CHEMBL1594435	-4.67	-3.86	CHEMBL1553297	-0.49	2.58	CHEMBL1623486	4.21	-0.67
CHEMBL1608121	-4.64	-11.26	CHEMBL1620926	-0.49	0.75	CHEMBL1463254	4.23	2.66
CHEMBL1408952	-4.59	-6.11	CHEMBL1864066	-0.48	-0.27	CHEMBL1495358	4.27	3.10
CHEMBL1364590	-4.55	-4.36	CHEMBL1401915	-0.45	3.08	CHEMBL1431360	4.27	0.09
CHEMBL1459983	-4.53	-7.70	CHEMBL1315510	-0.42	-1.55	CHEMBL1372201	4.29	-4.17
CHEMBL1312372	-4.52	3.10	CHEMBL1353857	-0.42	-1.08	CHEMBL416918	4.30	-0.17
CHEMBL1451534	-4.50	-2.72	CHEMBL1355793	-0.42	2.28	CHEMBL1473136	4.32	16.18
CHEMBL1904211	-4.50	1.95	CHEMBL2024322	-0.41	-7.62	CHEMBL1365336	4.33	1.80
CHEMBL1466811	-4.49	-1.31	CHEMBL1323047	-0.40	1.35	CHEMBL1301691	4.34	2.92
CHEMBL185823	-4.46	-1.29	CHEMBL1539035	-0.40	0.56	CHEMBL1521319	4.44	5.90
CHEMBL1546467	-4.41	-4.79	CHEMBL594864	-0.39	0.92	CHEMBL1326751	4.45	1.18
CHEMBL1879113	-4.40	-3.03	CHEMBL1582233	-0.33	-7.45	CHEMBL1604452	4.48	-0.57
CHEMBL1904485	-4.38	-8.31	CHEMBL1477092	-0.33	-2.52	CHEMBL2354932	4.51	-2.15
CHEMBL1877598	-4.38	-4.77	CHEMBL1618845	-0.33	1.54	CHEMBL1706053	4.52	3.47
CHEMBL1557321	-4.35	-5.44	CHEMBL1553653	-0.32	3.11	CHEMBL1379721	4.58	0.06
CHEMBL1592296	-4.35	-8.77	CHEMBL558103	-0.32	0.97	CHEMBL1492105	4.58	5.38
CHEMBL1491125	-4.31	-4.24	CHEMBL1372899	-0.32	6.70	CHEMBL1465921	4.75	7.16
CHEMBL1433159	-4.30	-2.53	CHEMBL1343555	-0.31	-1.81	CHEMBL1374188	4.78	6.53
CHEMBL1491180	-4.28	-1.05	CHEMBL1894885	-0.30	5.40	CHEMBL1336795	4.84	-0.10
CHEMBL1367196	-4.28	-5.22	CHEMBL1698075	-0.28	-1.04	CHEMBL1872689	4.87	7.58
CHEMBL1437664	-4.27	-4.03	CHEMBL1429784	-0.28	7.09	CHEMBL1624800	4.97	-2.33

CHEMBL1605783	-4.21	5.77	CHEMBL1565146	-0.27	0.42	CHEMBL1338081	5.03	7.36
CHEMBL1504992	-4.20	-2.26	CHEMBL577208	-0.25	-1.35	CHEMBL1377541	5.04	-19.80
CHEMBL462059	-4.20	0.03	CHEMBL1445809	-0.24	-0.99	CHEMBL1616526	5.10	8.82
CHEMBL401153	-4.18	-2.66	CHEMBL1448816	-0.23	1.73	CHEMBL2132995	5.11	1.29
CHEMBL1469050	-4.17	-8.21	CHEMBL2130815	-0.22	-1.43	CHEMBL588589	5.13	6.24
CHEMBL1439046	-4.15	2.65	CHEMBL1352711	-0.19	4.18	CHEMBL3126114	5.18	4.54
CHEMBL1372436	-4.14	3.17	CHEMBL1898941	-0.18	3.85	CHEMBL38181	5.25	2.73
CHEMBL1598316	-4.13	0.63	CHEMBL1418047	-0.18	-0.32	CHEMBL1450403	5.28	0.35
CHEMBL1602429	-4.12	0.49	CHEMBL1418841	-0.17	7.48	CHEMBL1389245	5.30	7.04
CHEMBL2354670	-4.11	-3.08	CHEMBL1210982	-0.16	7.60	CHEMBL259972	5.32	12.43
CHEMBL1332315	-4.09	-8.42	CHEMBL1431255	-0.12	-0.19	CHEMBL301522	5.39	5.56
CHEMBL1490702	-4.08	-2.01	CHEMBL1365293	-0.09	3.34	CHEMBL1540756	5.39	3.22
CHEMBL2358531	-4.02	3.19	CHEMBL1346130	-0.08	0.64	CHEMBL1593379	5.41	2.90
CHEMBL223235	-4.02	4.51	CHEMBL1603484	-0.05	-0.83	CHEMBL1443531	5.57	6.20
CHEMBL1596243	-4.00	-1.87	CHEMBL1709813	-0.04	-3.75	CHEMBL1885748	5.65	7.53
CHEMBL578066	-3.99	-12.07	CHEMBL1362106	-0.04	-1.05	CHEMBL1462688	5.67	7.67
CHEMBL1619955	-3.98	-2.07	CHEMBL1314627	-0.04	-0.19	CHEMBL1624735	5.75	2.98
CHEMBL1387772	-3.98	0.37	CHEMBL548113	-0.02	0.76	CHEMBL1433063	5.77	6.63
CHEMBL1312791	-3.95	-0.24	CHEMBL1355417	-0.01	0.86	CHEMBL1422616	5.78	-2.08
CHEMBL1348400	-3.92	6.28	CHEMBL1740954	-0.01	2.45	CHEMBL1573891	5.85	11.26
CHEMBL1885461	-3.90	-3.66	CHEMBL1623988	0.00	-2.52	CHEMBL1612075	5.86	7.44
CHEMBL1565136	-3.87	-5.32	CHEMBL1335461	0.00	4.56	CHEMBL1611755	5.93	4.17
CHEMBL1708694	-3.86	2.63	CHEMBL1415950	0.01	-0.01	CHEMBL81206	6.00	7.02
CHEMBL1430764	-3.78	1.63	CHEMBL1372765	0.03	-0.25	CHEMBL3289488	6.02	9.92
CHEMBL1891437	-3.77	2.16	CHEMBL2142523	0.10	-7.81	CHEMBL1715100	6.13	0.13
CHEMBL1492317	-3.76	0.02	CHEMBL1470168	0.10	1.57	CHEMBL1349356	6.28	4.98
CHEMBL392086	-3.72	2.00	CHEMBL1414038	0.12	-8.35	CHEMBL1517983	6.29	-1.32
CHEMBL1381653	-3.70	-3.88	CHEMBL1896668	0.13	1.67	CHEMBL1619666	6.30	5.31
CHEMBL3137465	-3.68	-0.12	CHEMBL1524989	0.14	4.42	CHEMBL1457888	6.39	-3.11
CHEMBL1543817	-3.65	-2.47	CHEMBL1617143	0.14	0.29	CHEMBL1545213	6.42	11.38
CHEMBL20526	-3.63	0.24	CHEMBL1461246	0.17	0.56	CHEMBL1368855	6.50	14.38
CHEMBL1586100	-3.62	1.20	CHEMBL1526274	0.18	0.39	CHEMBL1578452	6.53	-1.03
CHEMBL1715430	-3.61	-2.50	CHEMBL1712317	0.19	-1.38	CHEMBL1508158	6.56	1.30
CHEMBL600131	-3.60	3.19	CHEMBL1872168	0.19	3.15	CHEMBL1304465	6.60	0.10
CHEMBL1603425	-3.60	1.32	CHEMBL1734689	0.20	-0.98	CHEMBL1718316	6.66	5.97
CHEMBL1517506	-3.58	0.40	CHEMBL1475188	0.21	1.73	CHEMBL1517863	6.80	6.48
CHEMBL1388810	-3.54	-1.13	CHEMBL1452820	0.21	1.28	CHEMBL1308856	6.86	1.70
CHEMBL1592176	-3.53	-1.05	CHEMBL1463766	0.23	1.55	CHEMBL1736097	6.92	0.60
CHEMBL2136784	-3.53	2.88	CHEMBL2376451	0.25	-4.72	CHEMBL1546372	7.36	9.27
CHEMBL1500119	-3.52	1.21	CHEMBL1585133	0.25	0.62	CHEMBL1495	7.37	-0.32
CHEMBL2142374	-3.49	-0.83	CHEMBL1492555	0.25	2.31	CHEMBL1797399	7.48	0.96
CHEMBL132623	-3.49	2.59	CHEMBL1335177	0.27	-3.57	CHEMBL2133495	7.73	6.87
CHEMBL1550062	-3.48	-15.70	CHEMBL2158685	0.32	6.10	CHEMBL1308058	8.11	1.91
CHEMBL1489407	-3.41	0.14	CHEMBL401525	0.34	4.77	CHEMBL1490563	8.34	1.03
CHEMBL2354911	-3.40	-0.30	CHEMBL37072	0.35	2.00	CHEMBL1334595	8.37	5.37
CHEMBL1390356	-3.37	-6.63	CHEMBL1344315	0.36	7.52	CHEMBL1619006	8.71	6.58
CHEMBL1882461	-3.36	-5.66	CHEMBL1390653	0.36	5.75	CHEMBL1438039	9.19	4.72
CHEMBL1622517	-3.36	1.61	CHEMBL1451692	0.37	0.13	CHEMBL1485221	9.35	4.21
CHEMBL1597769	-3.35	1.58	CHEMBL233574	0.37	2.57	CHEMBL2144557	9.80	9.70

CHEMBL1905919	-3.34	-1.06	CHEMBL1475313	0.38	0.00	CHEMBL1345896	10.14	6.95
CHEMBL1369761	-3.34	-0.26	CHEMBL1355805	0.38	-1.88	CHEMBL1529042	10.47	9.75
CHEMBL1616511	-3.31	3.07	CHEMBL1517457	0.40	3.42	CHEMBL16850	11.37	4.34
CHEMBL1970527	-3.31	-4.33	CHEMBL1517175	0.41	4.37	CHEMBL1388546	12.15	6.88
CHEMBL1530929	-3.27	-1.13	CHEMBL2143234	0.44	1.86	CHEMBL1537539	12.24	6.37
CHEMBL1601974	-3.26	3.38	CHEMBL1570564	0.45	0.68	CHEMBL1544857	12.47	15.21
CHEMBL1703467	-3.25	-2.39	CHEMBL1411672	0.46	6.71	CHEMBL1349158	13.03	-1.75
CHEMBL1543151	-3.24	6.22	CHEMBL1624669	0.48	0.88	CHEMBL1315347	13.05	4.88
CHEMBL1624502	-3.24	1.01	CHEMBL1442345	0.51	1.00	CHEMBL1433682	16.00	14.01
CHEMBL259848	-3.22	3.57	CHEMBL1310704	0.52	4.32	CHEMBL2142076	19.10	19.85
CHEMBL1406896	-3.22	-9.40	CHEMBL1403960	0.54	1.17	CHEMBL1309267	23.31	26.07
CHEMBL1390179	-3.18	0.17	CHEMBL591020	0.55	-1.34	CHEMBL1395174	25.24	25.20
CHEMBL1563896	-3.17	-2.20	CHEMBL29213	0.55	1.49	CHEMBL597859	27.08	27.28
CHEMBL1488511	-3.14	8.94	CHEMBL407246	0.55	0.13	CHEMBL50024	29.14	30.00
CHEMBL1617807	-3.12	-4.71	CHEMBL246648	0.59	8.16	CHEMBL2361846	37.76	35.79
CHEMBL1607468	-3.09	-1.79	CHEMBL3186128	0.60	0.18			

6. Fig. S1: Hit CHEMBL1081548 activity confirmation in WDR5 HTRF

Dose response curves fitted from HTRF assay for CHEMBL1081548. IC50 = 75 μM with 50% inhibition in WDR5 HTRF and no inhibition in the counter assay.

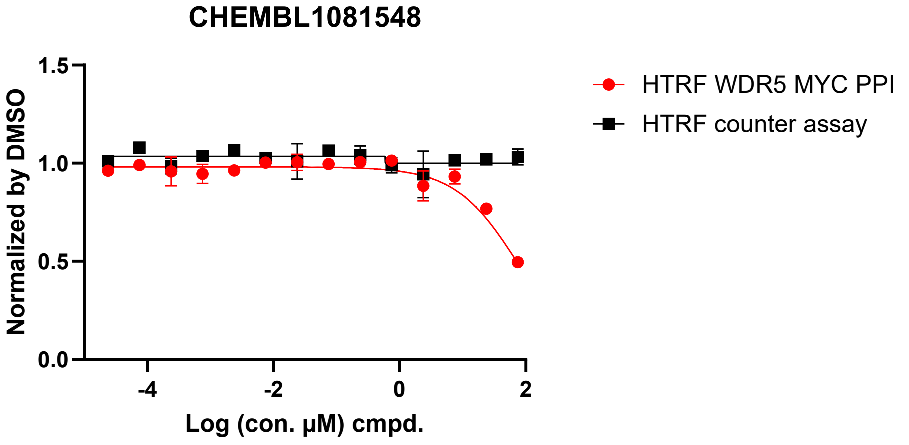

Supplement: Supplementary file 1 — Additional file 1: Pocket Crafter workflow process sample code and example dataset (ChEMBL). SMILES Molecular formula strings. Table S1. Protein Ligand Interaction Fingerprints (PLIF) summary. Table S2. Novartis diverse library HTRF screen result dataset. Table S3. ChEMBL compound set screen HTRF result. Figure S1. Hit CHEMBL1081548 activity confirmation in WDR5 HTRF. [file 13321_2024_829_MOESM1_ESM.pdf]
